# Review of Precision Medicine and Diagnosis of Neonatal Illness

**DOI:** 10.3390/diagnostics15040478

**Published:** 2025-02-16

**Authors:** Safaa ELMeneza, Naglaa Agaba, Rasha Abd El Samad Fawaz, Salwa Samir Abd Elgawad

**Affiliations:** Pediatrics Department, Faculty of Medicine for Girls, Al-Azhar University, Cairo 11651, Egypt; naglaafathi778@gmail.com (N.A.); rasha85@azhar.edu.eg (R.A.E.S.F.); salwa.samir@azhar.edu.eg (S.S.A.E.)

**Keywords:** precision medicine, neonatal brain injury, neonatal hemodynamics, neonatal RDS, neonatal sepsis, hyperbilirubinemia, AKI, BPD, pharmacokinetics, NICU

## Abstract

**Background/Objectives**: Precision medicine is a state-of-the-art medicine tactic that tailors information about people’s genes, environment, and lifestyle to aid the prevention, diagnosis, and treatment of various diseases to provide an overview of the currently available knowledge and applicability of precision medicine in the diagnosis of different cases admitted to the NICU, such as encephalopathies, respiratory distress syndrome of prematurity, hemodynamic instability, acute kidney injury, sepsis, and hyperbilirubinemia. **Methods**: The authors searched databases, such as PubMed and PubMed Central, for the terms neonatal “precision medicine”, “personalized medicine”, “genomics”, and “metabolomics”, all related to precision medicine in the diagnosis of neonatal illness. The related studies were collected. **Results**: The review highlights the diagnostic approach that serves to implement precision medicine in the NICU and provide precision diagnosis, monitoring, and treatment. **Conclusions**: In this review, we projected several diagnostic approaches that provide precision identification of health problems among sick neonates with complex illnesses in the NICU; some are noninvasive and available in ordinary healthcare settings, while others are invasive or not feasible or still in ongoing research as machine learning algorithms. Future studies are needed for the wide implementation of artificial intelligence tools in the diagnosis of neonatal illnesses.

## 1. Introduction

Precision medicine (PM) is the ability of medicine tactics that tailor information about people’s genes, environment, and lifestyle to aid the prevention, diagnosis, and treatment of various diseases. Precision medicine helps healthcare providers to expect which management strategies will be valid for specific populations. Precision medicine aims to provide more accurate prevention of diseases and point to the diagnosis mainly based on the genetics or molecular interpretation of the disease. Furthermore, it focuses on the appropriate treatments for “the exact patients at the accurate time”. Precision medicine has several advantages; among these, it facilitates recognizing the pathophysiological aspects of the disease and improves the use of specific treatments that best fit individual patients [1,2].

The U.S. National Research Council states that personalized medicine is an older term that refers to the same concept as precision medicine and carries the same meaning. However, personalized medicine is distinct in some ways, as the term personalized medicine infers treatments and prevention approaches adapted to the individual. Traditionally, the application of personalized medicine has broader aspects [3]. Precision medicine was established in the late 1990s; still, neonatal medicine has been late in applying PM.

Newborn infants admitted to the neonatal intensive care unit (NICU) suffer from complex diseases with myriads of different pathologies related to several systems and organs, such as respiratory distress syndrome (RDS), respiratory failure, and hemodynamic instability, due to several causes, such as patent ductus arteriosus and persistent pulmonary hypertension of neonates (PPHN). They are also subjected to acute kidney injury (AKI), electrolyte disturbances, encephalopathy and brain injury, seizures, hyperbilirubinemia, and sepsis. Neonatal guidelines for the majority of diseases are available, which could be beneficial for some sick neonates, but not for others. Subsequently, the strategy of one-size-fits-all is not always useful. Newborn infants may present with complicated disorders related to immaturity, as well as complications due to multiple organ dysfunction. Neonatologists may face difficult challenges in predicting the specific diagnosis and treatment of such infants. Hence, the role of precision medicine will help implement a well-organized plan of care, improving the prognosis and preventing serious outcomes and complications.

Precision medicine has recently emerged and utilizes huge amounts of biological data, including genomics, transcriptomics, epigenomics, proteomics, metabolomics, and pharmacogenomics. These diagnostic tools provide patients with effective treatment related to their genetic, biological, and clinical features [3].

Although some diagnostic tools are used in daily practice, those such as genomics and metabolomics are still restricted in terms of research due to a lack of current knowledge on neonatology. Conversely, non-invasive tools, such as NIRS, portable ultrasounds, and functional echocardiography, have been shown to support diagnosis and guide the management of sick neonates in the NICU [4,5,6].

The aim of this review is to present an overview of the currently accessible knowledge of precision medicine in the diagnosis of various cases admitted to the NICU, such as encephalopathies, respiratory distress syndrome of prematurity, hemodynamic instability, acute kidney injury, sepsis, and hyperbilirubinemia.

## 2. Information Sources

The authors looked at known databases, such as PubMed and PubMed Central, applying the phrases “precision medicine”, “personalized medicine”, “genomics”, and “metabolomics”, relating to precision medicine in the diagnosis of neonatal RDS, precision medicine in the diagnosis of neonatal sepsis, precision medicine in the diagnosis of seizures, precision medicine in the diagnosis of neonatal sepsis, and precision medicine in the diagnosis of hemodynamic instability and neonatal encephalopathy.

## 3. Neonatal Human Genome and Precision Medicine

The neonatal genome projects applied next-generation sequencing (NGS) in order to recognize curable pediatric onset genetic diseases, which facilitates early diagnosis, permits precise drug usage, and prevents complications [7]. Furthermore, the BabySeq project revealed differences between healthy and sick neonate genomic sequencing. Subsequently, sick neonates may need further analysis to identify the specific treatment associated with their clinical features and pharmacogenomic variants [8]. Overall, 25% of genetic disorders related to death in the NICU could receive treatment if diagnosed early [9].

The intent of the genomic sequencing in the acutely ill neonates project was to evaluate the usefulness of rapid, next-generation sequencing versus the existing diagnostic practice and treatment guidelines, as well as the prognostic data in acutely ill neonates and infants. It was reported that whole-genome sequencing (WGS) and rapid whole-genome sequencing (rWGS) may improve the diagnosis compared with regular genetic testing [10]. These sequencing techniques seem to have a beneficial value, as they encompass millions of tests at a time instead of conducting multiple tests. WGS determines the arrangement of all the nucleotides in human DNA and can detect variants within the genome regions [11].

The identification of gene variants may aid in the discerning of neonates at risk of severe disorders. Moreover, it could assist in terms of prevention and providing specific treatment. Nevertheless, there are limitations to WGS, as it cannot diagnose some disorders, such as congenital hypothyroidism, and is an expensive technique, especially in developing countries. Furthermore, there are some ethical concerns related to affected families.

Precision medicine through genetic studies can assess the risk of early complex diseases that neonatologists face daily. These complex diseases are affected by genetics and environmental factors. Distinguishing the genetic knowledge that contributes to the vulnerability, seriousness, and complications of these diseases will ensure early diagnosis and better outcomes. Variants in brain-derived neurotrophic factor (BDNF) have been connected with the grade of retinopathy of prematurity [12]. The polymorphisms in the high mobility group box 1 protein (HMGB1) have been related to the necrotizing enterocolitis predisposition, as well as to survival [13]. The risk of neonatal sepsis has been related to immature immune mechanisms, environmental and maternal factors, and neonatal genomics [14].

The integration of genotypic data from neonates with clinical data could predict the probability of bronchopulmonary dysplasia [15]. Moreover, the genotype and phenotype can be utilized for the appraisal of the hazards of drug use among neonates. The polygenic risk score (PRS) depends on data from genome-wide association studies (GWAS) and is composed of risk alleles, a risk magnitude for each allele, as well as the prevalence of the diseases. Therefore, PRS can assess the risk for each patient [16]. Moreover, it highlights preventive intrusions, suggests the age of onset, recommends lifestyle modifications, and evaluates the family risk of the diseases. However, PRS could cause false positive results [7].

Artificial intelligence, such as machine learning (ML), may be used to merge and organize the genotypes and phenotypes for the appraisal of the possibility of diseases.

## 4. Brain Injuries

***i.***  ***Early Brain Injury in Neonates and Neonatal Encephalopathy***

Early brain injury may be related to different categories, such as maternal comorbidities, placental abnormalities, encephalopathy, whether hypoxic ischemic (HIE) or non-HIE, and vascular incidents such as IVH, especially among preterm infants. Furthermore, brain injuries may be related to coagulopathy, such as arterial ischemic stroke, periventricular venous hemorrhagic infarction, or trauma. It may be associated with perinatal infections, such as cytomegalovirus, rubella, and chickenpox, or toxoplasmosis, jaundice, metabolic disorders, or genetic/epigenetic anomalies. These diseases may be fatal or lead to permanent brain damage or cerebral palsy.

Neonatal encephalopathy (NE) is among the most important reasons for admission to the NICU, mainly among full-term infants. The clinical definition of NE is a “clinical syndrome of disturbed neurologic function in the first week after birth in an infant born at or beyond 35 weeks of gestation, manifest by a subnormal level of consciousness or seizures, often accompanied by difficulty with initiating and maintaining respiration, and depression of tone and reflexes” [17,18]. The term HIE is usually used as being synonymous with NE; however, HIE is just one of the subclasses of NE. NE may be due to other causes rather than perinatal hypoxia-ischemia. NE is interrelated with multiple risk factors, such as sepsis, trauma, or vascular events [19]. Neonatal HIE is identified as the predominant risk for brain injury, responsible for 50 to 80% of cases admitted to NICUs [20].

A definite final diagnosis of HIE is based on retrospective data that include the suggestive history and nature of the neurological impairment. The neonatal diagnostic manifestation of HIE is presumptive, and the ACOG-AAP task force proposes it includes the following: a low Apgar score < 5 at 5 and 10 min; fetal umbilical acidemia (pH < 7.0 or base deficit ≥ 12 mmol/L); and a history of an abnormal fetal heart rate before birth and confirmation by the radiological verification of brain damage by MRI or magnetic resonance spectroscopy. Multi-organ dysfunction is one of the indicative diagnostic criteria for acute peripartum or intrapartum hypoxic or ischemic events that take place immediately before or during labor. The evolving outcomes may include spastic quadriplegia or dyskinetic cerebral palsy [17].

Although it seems to be decisive, the ACOG-AAP diagnostic criteria may not be specific enough to exclude other causes of NE, such as sepsis, trauma, or vascular events, or to include all HIE cases either. Moreover, there may be a combination of several risk factors, subsequently leading to the inadequate management of non-HIE causes. Furthermore, the previous diagnostic criteria do not specify the timing, duration, extent, or magnitude of the severity of the hypoxic insult, or consider the individual’s response to the precipitating event. It does not adequately stratify the grades of HIE. The reliance on the diagnosis of the mild stage by Sarnat and Sarnat classification affected decisions pertaining to therapeutic hypothermia (TH). Unfortunately, 25% of those diagnosed with mild HIE had abnormal neurological/developmental outcomes and cerebral palsy [21]. It was shown that the AAP-ACOG definitions could miss certain patients with HIE due to the non-inclusion of some aspects, such as milder acidosis or higher Apgar scores [22,23], typical MRI diagnostic criteria, or a progressive, unusual course of HIE [24]. Subsequently, the final diagnosis may not be finalized until a neurodevelopmental delay or impairment is seen later in affected infants or children.

Research by Lee et al. stated that a non-negligible percentage of HIE was due to impaired blood flow or oxygen delivery during birth, as around 50% of the studied cases had no clinical or radiological evidence of perinatal asphyxia [25]. Non-HIE NE causes can be misinterpreted as HIE at birth. Respiratory distress and abnormal muscle tone are common in congenital neuromuscular disorders, such as congenital myotonic dystrophy. Metabolic and/or genetic abnormalities may also manifest early, presenting with altered consciousness, seizures, and breathing difficulties [19].

A definitive early-precision diagnostic criterion for the different causes of NE is still needed to discriminate the wide range of diseases and abnormalities that cause NE [26]. The distinction between subclasses of NE at birth is difficult. Early signs and symptoms may be similar for diverse etiological diseases. The advancement in TH and other neuroprotection therapies, as well as the need for the early support of specific therapy for non-HIE encephalopathy, such as metabolic causes or rapid interference for surgical causes, necessitates an urgent individualized approach to save newborn infants from permanent brain damage.

As a result of all of these limitations, overdiagnosis, underdiagnosis, or misdiagnosis of HIE, as well as other causes of brain injury, may ensue. Each neonate’s life and quality of life matter to their family and society. This emphasizes the need to personalize precise data pertaining to the various risk factors, symptoms/signs, nature, and sites of brain damage to plan precision medicinal therapy.

Advances in PM may allow for precise diagnosis and offer proper, on-time, adequate neuroprotection therapy based on definitive individualized needs during high brain plasticity. Precision medicine may permit the recognition of the risk factors, grade of insults, and magnitude of the severity of the brain injury based on interpreting the results of ultrasounds, MRIs, EEGs, genetic profiles, and developmental milestones. Additionally, the ongoing studies and research that are currently taking place should be encouraged and supported to assist in the implementation of precision medicine in the NICU.

The current review presents some recent promising research related to prognosis and management (PM) in the area of brain injury. These studies show the rules of precision medicine in the anticipation, recognition, prevention, and reinforcement of appropriate decisions for neonates with brain injury in the NICU.

Genetic testing can help diagnose HIE and other causes of neurodevelopmental disorders by identifying genetic and epigenetic abnormalities. Additionally, it can provide visions of the severity and extent of brain damage and inform neuroprotective options. Genetic testing using targeted gene panel sequencing can identify the cause of NE and associated hypoxic brain injury, regardless of the primary clinical presentation. Lee et al., 2024, reported that 32.4% of the studied cases presented pathogenic variants that involved nine genes: CACNA1A, KCNQ2, SCN8A, STXBP1, NSD1, SCN2A, PURA, ZBTB20, and ENG [25].

In the personalized treatments developed according to the data of the studied genetic tests, the sodium-channel blockers for neonates with KCNQ2 or SCN8A variants and the use of a ketogenic diet in cases with STXBP1 or SCN2A mutations, these therapies showed particular usefulness among the studied cases [25].

Zhang et al., 2024, studied the magnified mutation range of genes related to NE and provided new validation for molecular diagnosis in unexplained neonatal encephalopathy. They reported 15 NE-related pathogenic variants. A total of 7 of the pathogenic variants related to the identified 12 genes were related to premature translation termination, and 4 variants were destructive to the protein structure of KCNQ2 [27]. A cohort study by Yang et al., 2020, identified 30 genes for NE. Epileptic encephalopathy represented the major gene ratio (58.5%), followed by metabolic and syndromic (18.9%) and then the mitochondrial, in 3.8% of the studied cases. The most involved genes were epileptic-related genes, KCNQ2 and SCN2A. There were 10 genes recognized to be encephalopathy-causing genes, comprising KCNQ2, SCN2A, SCN1A, KCNT1, CDKL5, STXBP1, and ARX, related to epileptic encephalopathy, SERAC1, and AMT linked to metabolic encephalopathy, as well as MECP2 in syndromic encephalopathy [28]. It has been shown that epileptic genes, ALDH7A1, DEPDC5, and PRRT2, metabolic genes, DBT22 [29] and MMACHC [30], a mitochondrial gene, NDUFA11 [31], and nine syndromic genes cause diverse brain abnormalities and/or nervous system damage and/or encephalopathy. A study by Montaldo et al., 2019, showed marked differences in the gene expression profiles of neonates with neonatal encephalopathy at the time of birth in response to hypoxia compared with the healthy controls and septic neonates [32].

Accordingly, genetic testing may empower personalized neuroprotective treatment if a known specific variant is identified and a known target therapy is available too. A study of the transcriptomic profile of adverse neurodevelopmental outcomes after neonatal encephalopathy disclosed the marked expression of genes associated with melatonin and polo-like kinase pathways in neonates with adverse outcomes. It was proposed that transcriptomic profiling could help with the prompt risk grading of NE [33].

There were significant differences in the genome expression profile during the first three days following birth between newborn infants with HIE from developed countries and neonates from South Asia. This may explain the inadequate response to TH among infants in developing countries. The adverse outcome was related to the attenuation of eukaryotic translation initiation factor 2 in the high-income countries’ neonates, while it was related to aldosterone signaling in epithelial cells in the South Asia neonates [34]. Subsequently, according to this study, the whole-blood genome expression profile will possibly be beneficial for diagnosis and personalized neuroprotection in HIE, as well as for observing the response to therapeutic remedies. However, we have to look at race differences when evaluating the differences in response to therapy. In addition, future research will help to elucidate the link between genetic variation in categorizing NE or screening the response to therapy.

Artificial intelligence (AI) has been applied in research to add value to neuro-precision medicine. The machine learning algorithm (ML) was used and validated for recognizing neonates at risk of HIE by Murray et al., 2024. The algorithms were applied to compute a probability index for the occurrence of HIE. The model may assist in early diagnosis and hasten the recognition of neonates who require continuous neurological or neurophysiological monitoring [35]. The ML models using MRI findings were shown to be able to anticipate neurodevelopmental consequences in newborn infants with hypoxic-ischemic encephalopathy throughout all neurodevelopmental domains [36]. A detailed MRI scoring system allows for the prognostic appraisal of motor, cognitive, and composite sequences. Damages in the deep grey matter and cerebellum were prognostic of unfavorable outcomes [37].

Recent ML models of cerebral oxygenation (rcSO2) for the detection of brain injury in term neonates with HIE have more success, as the algorithm can merge a selection of features to predict the outcomes rather than one quantifiable approach to summarize the signal. It is based on automated models of NIRS. This model looks at the rcSO2 features and their relation to the severity of HIE, classified by a modified Sarnat score at 1 h. One such study concluded that the automated analysis of regional cerebral oxygen saturation, rcSO2, using either ML or deep learning methods, was able to identify infants with adverse outcomes [38].

Low rcSO2 could indicate hypoxia, decreased blood flow, vasoconstriction, and/or abnormal cerebral autoregulation.

An ML model was developed using a combination of early EEG background analysis and clinical data to support the expectation of neonates with HIE who were at the highest risk of seizures, hours before the seizures appeared. This reinforced the application of automated measurement tools for the early assessment of HIE [39]. Another study showed the role of automated models for the diagnosis of neonatal encephalopathy by applying aEEG deep neural networks. The researchers confirmed that deep neural network methods may advance the precision identification of neonatal encephalopathy, particularly for describing the EEG background pattern, sleep-wake cycling, and seizures that help neonatologists attain a precision diagnosis [40].

However, the diagnostic role of machine-learning methods has to be proven with more studies and large cohorts of neonates. Some studies need to overcome limitations, such as the stage of the illness, the timing of the performance of ultrasounds or MRIs, the timing of the incident of IVH, and the synchronization of the measurements of SPO2 and rcSO2 [41].

Metabolomics provides the metabolic status of cells, tissues, or organisms concerning genetic variations or external stimuli. Metabolomics patterns may be valuable in the early detection of perinatal asphyxia-induced HIE. It could aid in planning individualized neuroprotective and therapeutic hypothermia (TH) strategies [42]. The metabolite 2-phosphoglyceric acid is decreased in OGD/R-induced HT-22 cells and participates in the neuroprotection of OGD/R-caused cell demise and HIBD-provoked neuronal injury. Applying 2-phosphoglyceric acid as a therapeutic agent decreases brain injury and neuronal damage by downregulating Acyl-CoA synthetase long-chain family member 4 and increases glutathione peroxidase 4 expression. It has been suggested that 2-phosphoglyceric acid could offer promising neuroprotection in brain damage due to HIE [43]. The study of metabolomics is promising for individualized therapy.

Accordingly, recent research studies have helped to modernize neonatal practice in implementing personalized and precision medicine for neonatal neurologic illness. The previously mentioned studies were concerned with further developing new methods based on ML models and the automated exploration of data, controlled by experts who applied MRI, NIRS, and EEG, as well as genetic studies and omics. These new tools may offer a fast, effective method for the prediction, diagnosis, and categorization of brain injury. Nevertheless, further studies are still needed to validate the efficacy of these tools with a larger cohort of neonates to support their future implementation for the prevention, diagnosis, and treatment of brain injury before it becomes permanent. Hopefully, these tools will be simple and friendly to use, particularly throughout the first hours following labor to protect the brains of newborn infants.

These new tools can support decisions pertaining to more precise diagnosis monitoring and therapy. Genetic testing could assist in the prediction of the possibility of insults and adverse outcomes and could differentiate between subclasses of NE and precise therapy. ML models can support decisions by monitoring the progress of illness and the prediction of outcomes, while metabolomics could support individualized therapy.

***ii.***  ***Therapeutic hypothermia***

Therapeutic hypothermia is the primary neuroprotective strategy applied clinically to neonates with a moderate degree to the highest degree of hypoxic-ischemic encephalopathy. It involves decreasing the infant’s core temperature to around 33–34 °C for the duration of the first 3 days after birth, which helps to reduce metabolic demand and limit brain injury [44,45]. The cooling should ideally start within the first 6 h after birth to maximize its effectiveness [45]. In the context of therapeutic hypothermia in neonates, personalized medicine aims to give individualized and targeted interventions depending on specific factors that can influence the effectiveness and safety of this treatment approach [46]. Precision medicine approaches may include patient selection and advanced and appropriate diagnostic tools. Cooling therapy is recommended for full-term or near-term infants with moderate-to-severe HIE. The specific criteria include a cord blood pH ≤ 7.0 or base deficit ≥ −16, or acidotic blood pH (7.01 to 7.15) with perinatal events of acute decrease in oxygen supply, and a low Apgar score ≤ 5 at 10 min [47]. The use of advanced diagnostic techniques includes neuroimaging cranial ultrasound (CUS), MRI, EEG, and near-infrared spectroscopy (NIRS) [48]. Moreover, biomarker analysis can accurately evaluate the degree of neuronal cell damage and predict treatment response. Incorporating genetic and biomarker analyses can help detect patients more likely to react positively to hypothermia, allowing for a more personalized approach [47]. Heart rate variability (HRV) is considered a promising biomarker for the prediction of the severity of electroencephalogram changes in neonates with HIE during the first 12 h of life, and the non-invasive hemodynamic monitoring of cardiac functions, e.g., stroke volume and cardiac output during therapeutic hypothermia and rewarming, can assess the severity of HIE [49]. Studying the kinetics of circulating progenitor cells (CPCs) in infants with encephalopathy can demonstrate the process of cellular repair after injury [50]. Utilizing machine learning algorithms and AI technologies to analyze large datasets can help predict outcomes and tailor interventions based on individual patient characteristics [47]. The monitoring and follow-up of neonates during TH by a multidisciplinary team for brain-oriented care is crucial, and they should follow specific protocols and be supplied with all of the diagnostic tools and the connected electronic health record (EHR) system [47,51]. It is important to implement comprehensive monitoring protocols and continuously monitor patients during and after hypothermia treatment to help identify complications early and adjust treatment as necessary [52]. In addition, 24 h electroencephalogram monitoring is recommended to diagnose seizures, as well as a proper follow-up of laboratory parameters, during the cooling period to guide adjustments in temperature management and optimize the therapeutic effects [53]. Long-term outcome tracking and establishing systems for follow-up of infants undergoing therapeutic hypothermia can provide valuable data on efficacy and safety, helping future practices [54].

Limitations and Gaps In the Knowledge for Precision Medicine in Therapeutic Hypothermia

The application of precision medicine in TH for neonatal HIE faces several challenges and gaps in the knowledge that can limit the effective implementation of tailored therapies that could improve outcomes for affected infants. There are several challenges regarding variability in terms of response. There is significant variability in how infants respond to therapeutic hypothermia; the post-conception age, the timing of the intervention, and the degree of HIE can influence outcomes, but the interplay of these factors has not been well characterized [55]. Furthermore, the gaps in the knowledge orientate around reliable biomarkers that can predict which infants will benefit most from therapeutic hypothermia. Current research has not yet established definitive biomarkers to effectively guide treatment decisions [56]. The available data emphasize the need to adjust patient selection and stratification for TH; tailoring treatment for these populations could enhance its efficacy [11]. There is a need for more research to study the variability in response to TH, such as biomarkers and genetic profiling, in addition to the appropriate implementation of AI and ML algorithms. High-quality trials that include diverse patient populations and standardized protocols are essential for validating the efficacy of therapeutic hypothermia.

Precision medicine is a promising approach in the context of therapeutic hypothermia in neonates and aims to provide individualized and targeted interventions based on specific factors that can influence the effectiveness and safety of this treatment approach, helping early recovery and reducing long-term disabilities.

***iii.***  ***Neonatal Seizures***

Neonatal seizures are among the most major clinical problems in the NICU. They may be too distinct to be the cause of admission, or they may be subclinical and detected through the monitoring of infants who have been admitted due to other diseases. Usually, a seizure is a sign indicative of brain injury [57]. Seizures are sudden, paroxysmal, repetitive, stereotypical events, and abnormal alterations of electrographic activity from birth to the end of the neonatal period [58,59]. An electrographic neonatal seizure is defined as a sudden, abnormal EEG event, defined by a repetitive and evolving pattern with a minimum 2 μV peak-to-peak voltage and a duration of at least 10 s. Seizures are either motor or nonmotor, whereas motor seizures are predominantly focal or multifocal and include automatisms, clonic, epileptic spasms, myoclonic, sequential, and tonic. Nonmotor seizures include automatisms and behavioral arrest [59,60].

The clinical identification of seizure morphology per se is not diagnostic in every infant; some signs may mimic normal behavioral activities or even have no physical signs at all, depending upon the etiology, grade of severity, and course of illness [61,62]. Severe HIE may not show clinical seizures. However, ictal activities may be seen in the EEG.

Severe illnesses in newborn infants, such as HIE, vascular stroke, hemorrhage, infection, hypoglycemia, or electrolyte imbalance, present with acute symptomatic seizures and account for a high percentage of neonatal seizures. Some respond to treatment of the related risk factors, such as hypoglycemia or hypocalcemia. However, some seizures do not respond to general management, such as neonatal epilepsy syndromes related to structural abnormalities, genetic syndromes, or inborn errors of metabolism. These seizures are still difficult to recognize and may evade early therapy [59]. Neonatal-onset epilepsies represent around 10–15% of neonatal seizures [63].

One of the challenges for the early diagnosis of neonatal seizures is the nature of the seizure. The majority of seizures are exclusively electrographic, while clinical seizures have no electrographic findings [64,65]. Subtle and subclinical seizures possibly may not be perceptible [66]. However, it has been suggested that seizure semiology could suggest the primary illness; focal clonic seizures are significantly associated with vascular stroke or infection, while focal tonic seizures are related to genetic epileptic encephalopathy. Autonomic seizures could be associated with bleeding, and myoclonic seizures could signify inborn errors of metabolism [67,68].

One of the diagnostic and monitoring tools in the NICU is the aEEG/EEG, as well as near-infrared spectroscopy. The aEEG is useful for detecting repeated seizures and status epilepticus, and the trend also provides instant and significant data regarding evolving brain functions over time and during sleep-wake cycling [69]. Conversely, a systematic review report revealed that the aEEG cannot be advocated as the only tool for recognizing seizures in newborn infants [70]. Barely 30% of one-time seizures can be perceived in the trend of an aEEG, but the precision of the trend increases when seizures are more recurrent and with longer durations [69]. Subsequently, from this data, multi-channel EEG recordings, conventional EEGs, and continuous video-EEGs are important for diagnosis, monitoring, and evaluating the efficacy of the therapy [71,72].

Genetic testing, such as whole-exome sequencing, may identify about 75% of the etiology of seizures in neonates with neonatal epilepsy [73,74], and whole-exome sequencing can avoid the phenotypic intersect of different genetic epilepsies [74,75]. Self-limited familial neonatal seizures that are AD can be differentiated by a genetic disorder in KCNQ2, KCNQ3, and SCN2A. Early-infantile epileptic encephalopathy associated with structural brain malformations is linked with genetic variants in ARX, CDKL5, SLC25A22, STXBP1, KCNQ2, SPTAN1, SCN2A, and metabolic disorders. Early myoclonic encephalopathy associated with metabolic disorders, genetic variants in STXBP1, TBC1D24, and GABRA1, and epilepsy of infancy with migrating focal seizures are related to pathogenic variants of KCNT1, SCN2A, SCN1A, SLC25A22, PLCB1, and QARS genes. Self-limited neonatal seizures have a favorable outcome and may disappear within 48 h; on the contrary, early-infantile epileptic encephalopathy has early-life mortality or severe developmental disabilities [59].

Other challenges in the diagnosis of neonatal seizures are represented by the seizure’s mimic movement, such as jitteriness, hyperekplexia, benign sleep myoclonus, rapid eye movement sleep behavior, apnea, various motor automatisms, and dystonic or tonic posturing provoked by stimulation [60,76,77]. Although it could be differentiated by abolishing with touch, repositioning the limb, or no abnormal changes in EEG, it may represent contributory disease or could be related to non-electrographic seizures [77].

Subsequently, precision diagnosis batteries have to include the EEG to detect electrographic activities and neuroimaging for recognizing disorders in the brain structure, involving hemorrhage, infarction, or defects of cortex development, as well as chemical tests for glucose and electrolytes. Additionally, genetic testing is essential to offering individualized treatment and achieving better neurocognitive development outcomes.

Precision diagnosis will direct specific therapy that suits the individual variability, such as phenobarbitone, fosphenytoin, phenytoin, and levetiracetam, for the treatment of acute symptomatic seizures or TH in HIE. Medication such as conventional sodium channel inhibitors is proposed for defects in SCN2A and SCN8A genes; on the contrary, it may worsen if given to cases with defects in the SCN2A gene. Pyridoxal phosphate is suitable for pyridoxal-phosphate-dependent epilepsy, with a low-purine or low-protein diet for molybdenum cofactor deficiency with cyclic pyranopterin monophosphate if the defect is in the gene MOCS1 [78].

The diagnostic and supervising methods appear to be insufficient to precisely predict or reinforce the diagnosis of seizures in the NICU. There is a need for a machine learning model to be developed and include the essential data, such as the gestational age, the age of onset of seizures, the category of seizure, the duration of seizures, the semiology, MRI and other neuroimaging, and the electrical activities of the EEG, to support the precision diagnosis of etiological causes of seizures and support therapy. Future studies are needed to support the complete care of neonates with seizures: What is a suitable screening method for the prediction and early detection of seizures in a busy NICU? What is the right medicine/therapy for this specific newborn infant? For how long should this medication be given?

## 5. Hemodynamic Disturbances

***i***  ***Patent Ductus Arteriosus (PDA)***

Precision medicine is important to ensure the management of the hemodynamic stability of neonates admitted to the NICU. Patent ductus arteriosus (PDA), hypotension, and shock, as well as persistent pulmonary hypertension of the neonates (PPHN), are among the common problems that require timely diagnosis and management.

The hemodynamic instability among these infants may be due to the response to stress factors, such as hypoxia, infection, hypoperfusion, immature physiological response, and inadequate compensatory mechanisms. Moreover, congenital heart disorders, mechanical ventilation, and medications such as chronotropic, analgesic, and muscle relaxants may also participate in hemodynamic instability. Usually, we depend upon indirect measures to monitor hemodynamics, such as heart rate, blood pressure, capillary refill time, urine output, and blood gases [79]. The mechanisms of instability and primary pathophysiology are not similar for each neonate. Subsequently, to improve the outcomes of these infants, personalized medicine aims to make a precise diagnosis of the underlying precipitating cause, followed by precise treatment medication and monitoring the effect of treatment.

The assessment of patent ductus arteriosus concerns the hemodynamic assessment of PDA using transthoracic echocardiography and is the gold standard for the diagnosis and monitoring of treatment; clinical signs only are undependable. Both 2D Doppler echocardiography-targeted neonatal echocardiography and cardiac point-of-care ultrasound can be used for diagnosis and therapeutic precision in the NICU [80]. Echocardiography reveals hemodynamic, significant PDA (hs PDA), and hence, the need for treatment. Echocardiography data display the shape, size, and volume of the shunt, as well as the pulmonary pressure and other structural cardiac lesions, including duct-dependent cardiac defects [5,81]. Neonatologists have proven that echocardiography enables a longitudinal assessment of the early recognition of PDA in preterm infants with high-flow ductal volume, hence assisting in planning for appropriate therapeutic interventions, such as drug dosages and intervals [5].

In preterm infants, it is important to estimate the volume of transductal left-to-right shunt to recognize those who will need immediate active medical therapy. If not treated, shunt may cause increased pulmonary blood flow and decreased systemic perfusion, which could be related to multiorgan comorbidities such as pulmonary hemorrhage, bronchopulmonary dysplasia, intraventricular hemorrhage (IVH), necrotizing enterocolitis (NEC), or focal intestinal perfusion, as well as brain injury periventricular leukomalacia and impaired school performance [79,82]. The assessment of PDA has to evaluate ductal characteristics, ductal size, magnitude, and the impact of the shunt assessment of pulmonary circulation and systemic hypoperfusion. PDA size is considered small when less than “1.5 mm, moderate from 1.5 to 1–2 mm, and large more than 2 mm”. Furthermore, evaluation determines the flow direction, left to right, right to left, or bi-directional, and Doppler assessment, maximum velocity (Vmax) in systole and end-diastole. The assessment of pulmonary over-circulation is recognized when the dilated left side of the heart and the LA/Ao ratio is considered mild when <1.4, moderate in the range 1.41–1.6, severe when >1.6, or when LVEDD or LPA diastolic velocity: mean velocity > 0.42 m/s, end-diastolic velocity > 0.2 m/s, or a reversal of the mitral E/A ratio. Moreover, to verify the significance of intra-atrial shunt, the markers for systemic hypoperfusion are retrograde or absent blood flow during diastole in the descending aorta, coeliac trunk, or superior mesenteric artery, or the anterior or middle cerebral artery [83,84]. However, some of these measurements have interobserver variability [85]. Generally, the diagnosis of the hs PDA has to include at least the following: (1) diameter of the ductus arteriosus > 2.0 mm; (2) ductal flow pattern (‘growing’ pattern or pulsatile with Vmax < 2 m/s and Vmax/Vmin > 2); (3) retrograde post-ductal aortic/coeliac/SMA diastolic flow; (4) La/Ao > 2; (5) LVO > 300 mL/kg/min; (6) Mitral valve E/A ratio > 1 [83].

Spontaneous closure of the duct is favored by some molecular, physiological, and structural factors. Some genetic factors are associated with an increased risk of PDA, such as TFAP2B (rs987237), TRAF1 (rs1056567), and AGTR1 (rs5186), while others, such as PTGIS (rs493694, rs693649), ESR1 (rs2234693), and IFN-g (rs2430561), are linked to decreasing the risk of PDA. The possibility of the “rs7557402 SNP” variant resulting in failure following ibuprofen treatment of PDA closure has been reported [82,83]. This genetic variance may explain the resistance to therapy in some cases.

Other diagnostic tools for PDA include platelet count and indices, cardiac peptides and near-infrared spectroscopy (NIRS), proteomics analysis, and machine learning prediction models [84]. Recent evidence proposes that low platelet counts and platelet dysfunction are associated with an unsatisfactory response to treatment [86]. Cardiac peptides, such as BNP/proBNP and cardiac high-sensitivity troponin T, are correlated with hs PDA [87,88]. Near-infrared spectroscopy is a noninvasive monitoring tool for regional tissue oxygenation and aids in preventing multiple organ comorbidity in other organs, such as the brain, kidneys, and intestines [89]. Lung ultrasound can also postulate early detection of hs PDA through the evaluation of pulmonary edema levels in extremely preterm infants [90].

The perfusion index (PI) is a simple, continuous parameter provided by pulse oximetry to assess the peripheral perfusion, and it could help to predict a significant PDA [89,91]. Electrical cardiometry/electrical velocimetry can be used in addition to echocardiography, as it is non-invasive, monitors the cardiac output, and has been proven to be safe and feasible [89].

Recent studies have shown proteomic analysis differences in 21 proteins and 8 cytokines between neonates with a large PDA and neonates without a PDA; there was an elevation in angiotensinogen, periostin, and pro-inflammatory associations, including interleukin (IL)-1β and IL-8, and anti-inflammatory associations, including IL-1RA and IL-10, while complement factors C8 and carboxypeptidases were decreased. One study reported an association between PDA and the renin-angiotensin-aldosterone system and immune and complement systems [92]. Another study showed that the level of plasma protein disulfide-isomerase A6 (PDIA6) was downregulated in patients with PDA; it may have potential clinical implications for PDA treatment and provide evidence regarding the etiology and molecular mechanism of PDA [93]. Proteomic studies may implicate a new insight into the pathogenesis of and approach toward the management of PDA, especially among refractory cases.

Machine learning has been developed to calculate the effectiveness of treatment for hs PDA closure. Deep learning and convolutional algorithm models identified PDA with a 0.84 positive predictive value, a 0.80 negative predictive value, 0.76 sensitivity, and 0.87 specificity [94]. ML was developed to enable the identification of the signature of sounds of PDA and congenital heart diseases with promising results [94]. A study by Park et al. showed that an AI-based PDA diagnostic support system had 84% accuracy and the ability to detect PDA symptoms up to 3.3 days in advance [95]. The implementation of AI-dependent models can assist in the early prediction of infants and provide on-time treatment; however, further studies are needed to include a dataset that can provide more accuracy for these models.

Treatment is directed toward hemodynamically significant PDA. Recently, it has been advised to monitor infants for the spontaneous closure of ductus arteriosus and avoid prophylactic treatment; a decision has to be made based on clinical, echocardiographic, and NIRS evaluations and to include the neonate’s gestational and postnatal ages. Pharmacologic therapy has been proposed for hs PDA and includes drugs that inhibit prostaglandin, such as cyclooxygenase inhibitors, indomethacin, and oral or intravenous ibuprofen and paracetamol (acetaminophen) [96].

However, there is an abnormal response to the pharmacological treatment of PDA due to genetic variances. The low clearance of indomethacin and ibuprofen is related to the cytochrome P450-related enzymes CYP2C9 and CYP2C8. No responsiveness to pharmacological treatment is related to genes associated with prostaglandin and NO action or synthesis, such as SLCO2A1, PTGS2, and NOS3 [97].

The precision diagnosis of PDA mainly depends on echocardiography, which can predict hs PDA and longitudinal assessment for the response to pharmacotherapy, and NIRS determines tissue oxygenation assistance to support other organs and facilitate the stabilization of the hemodynamics of the newborn in the NICU. Other diagnostic tools may play a role in the future, such as proteomics and machine learning tools, for the prediction of PDA. Treatment is reserved for hs PDA and should be based on the integration of the neonates’ personalized needs and clinical data in conjunction with echocardiography markers.

***ii.***  ***Neonatal Shock***

Sudden unexpected clinical deterioration or cardiorespiratory instability in neonates is often referred to as a “crashing” neonate. It is an apparent life-threatening event. Several risk factors are associated with crashing neonates: trauma (accidental and nonaccidental, such as abuse); heart disease (structural and nonstructural); hypovolemia/arrhythmias/hypoxia; endocrinopathies (congenital adrenal hyperplasia, thyrotoxicosis); metabolic abnormalities (electrolyte imbalances); inborn errors in metabolism; seizures; formula mishaps (mix-ups/under- or over-dilution); intestinal catastrophes (necrotizing enterocolitis, intussusception, and midgut volvulus) or omphalitis; toxins exposure/poisons; and sepsis (meningitis, pneumonia, and urinary tract infection).

Shock is a state when there is a discrepancy between oxygen need and delivery on a cellular level. Subsequently, physiologic compensatory mechanisms are initiated to overcome the insults, and according to the response, three shock phenotypes may be seen: compensated shock, uncompensated shock, and irreversible shock [82].

In neonates, the compensatory phase may pass unnoticed. Blood pressure could be normal or even high and not reflect the low cardiac output. This means that we cannot rely on blood pressure only to anticipate hemodynamic instability (HI) and shock in newborn infants. The precise diagnosis of shock is a must, as sick neonates have limited and immature compensatory mechanisms and may quickly end up with irreversible shock.

Thorough, cohesive hemodynamic monitoring using clinical data, such as blood pressure, pulse pressure, capillary refill time (CRT), oliguria, and NIRS, to assess regional tissue oxygenation and assess tissue oxygen saturation (StO2) and fractional oxygen extraction (FOE) will be helpful during diagnosis before the increase of lactic acidosis. Moreover, the evaluation of cardiac output by transthoracic echocardiography/targeted/neonatal performed echocardiography, as well as biomarkers of organ dysfunction such as lactic acid, will aid in the early individualized treatment of sick neonates in the NICU and avoid complications of low cardiac output [5,98]. Furthermore, cardiac output can be assessed with other tools, such as electrical biosensing technologies and transpulmonary ultrasound dilution [82].

Recently, point-of-care ultrasound (POCUS) has been integrated into complex clinical situations and emergencies in the NICU. The first step in the decision tree is ruling out cardiac tamponade, followed by pneumothorax and pleural effusion, and then acute critical aortic occlusion, acute abdominal complications, and severe intraventricular hemorrhage [99]. Another POCUS protocol was developed by Elsayed et al., 2023, in which experts take 10 min to screen life-threatening conditions. POCUS supports the individualized management of the complex pathophysiology of hemodynamic instability by assessing cardiac function, including myocardial performance, volume status, and systemic blood flow. POCUS differentiates between volume depletion from vasoconstrictor physiology or cardiogenic shock. Subsequently, neonatologists can support management precisely if volume depletion starts, and until vascular and heart filling is restored, inotropes should be avoided. However, in vasoconstrictor physiology or cardiogenic shock, inotropes are indicated [100].

However, POCUS screening cannot detect congenital heart diseases or offer a detailed assessment of pulmonary hypertension, ventricular functions, or arrhythmias, as it is not the scope of POCUS [100]. A new physiologic-based, integrated algorithm for the prediction and assessment of neonatal HI was published; this algorithm takes into consideration the heterogeneity of the different physiologic mechanisms that cause hemodynamic instability. HI was categorized into five classes based on blood pressure, echocardiographic markers, and oxygen indices, which include hemodynamic instability due to vasodilatory physiology, hemodynamic instability due to vasoconstrictive physiology, cardiogenic shock, volume depletion, and left-to-right shunt physiology. The algorithm incorporates a combination of the integrated monitoring markers to facilitate the diagnosis of the shock phenotype; for example, hemodynamic instability due to vasodilatory physiology can be predicted when there is a decrease in diastolic blood pressure or a trending down, decrease in systolic and mean blood pressure with normal pulse pressure, low systemic vascular resistance, brisk CRT, PI > 3, and oliguria or urine output < 1 mL/kg/h for at least 12 h beyond the first 12 h after birth. NIRS markers show an increase in end-organ oxygen extraction, which is a warning for compromised autoregulation and low oxygen delivery, and if amplified extraction moves to full capacity, lactic acidosis is an ominous sign of organ failure [101].

Using such algorithms enables individualized treatment for the shock phenotype according to the HI precipitating trigger. Regarding the previous example of vasodilatory shock, vasopressors such as norepinephrine, vasopressin and its equivalent, and steroids in resistant cases may be the correct specific therapy. If echocardiography shows an underfilling of both ventricles, anticipate volume depletion shock, and if the heart is dilated, consider the diagnosis of chronic venous congestion.

Integrated monitoring of the HI could decrease adverse events from using inotropes, vasodilators, or unnecessary fluids for vasodilatory shock or diuretics and inotropes in volume depletion shock, as well as inotropes or extra fluids in the case of cardiomyopathy with chronic venous congestion.

## 6. Respiratory Disorders

***i.***  ***Neonatal Respiratory Distress Syndrome***

Respiratory distress syndrome (RDS), or hyaline membrane disease, is the common cause of respiratory distress in preterm neonates caused by a deficiency in pulmonary surfactant due to either inadequate surfactant production or surfactant inactivation in the context of immature lungs. RDS can occur also in near-term and full-term infants due to genetic mechanisms disrupting surfactant metabolism, resulting in diffuse lung disease in near-term and full-term infants that mimics RDS in preterm infants. It is diagnosed clinically based on the onset of progressive respiratory insufficiency shortly after birth in a preterm neonate in conjunction with a characteristic chest radiograph [102].

The need for precision medicine approaches for both the diagnosis and treatment of neonatal RDS is essential, and continuous attempts to apply the same treatment to all RDS patients are unlikely to be useful. Therapeutic agents can be employed for the specific pathophysiology and actual patient requirements that are the future of neonatal RDS and likely to derive benefit [103]. Cortisol levels are correlated with the severity of RDS and can predict respiratory support strategies [104].

Precision medicine is based on tailoring patient information by combining the interaction of genetic predisposition, clinical information (e.g., gestational age, ethnicity, and gender), and individual biomarkers to provide a more precise stratification of different phenotypes within preterm infants [105].

There are three unique challenges in the implementation of precision medicine approaches in neonates with RDS:

- Surfactant genetic and biological tests;
- Advanced oxygenation metrics;
- Functional lung imaging.

Surfactant genetic and surfactant biology of RDS neonates

Pulmonary surfactant is a complex mixture of lipids and proteins that covers the inner lining of normal alveoli that are needed to prevent end-expiratory atelectasis. Surfactant is stored in specialized organelles in alveolar type 2 cells called lamellar bodies, storage particles consisting of packed surfactant phospholipids and proteins, released from type II alveolocytes before its secretion into the airspaces. The phospholipid components of surfactant, mainly disaturated phosphatidylcholine (DSPC), are responsible for lower surface tension, and these are imported into lamellar bodies by ATP binding cassette sub-family A, member 3 (ABCA-3). Surfactant deficiency can be caused by sequence variants in the gene (ABCA3), disrupting or limiting its production. The exact amount of functional ABCA3 needed to prevent lung disease is not known, and production is developmentally regulated, increasing with advancing gestational age [106].

Surfactant proteins B (SP-B) and C (SP-C) are low-molecular-weight hydrophobic proteins that have important roles in surfactant function and metabolism, as any genetic mechanisms disrupting or altering the production of SP-B and SP-C might result in a phenotype of RDS [107].

A summary of the specific genes and proteins and key clinical features is featured in Table 1.

Knowing the genetic mechanisms of RDS is useful for understanding the typical clinical presentations of neonatal RDS, establishing the diagnosis, and performing genetic counseling for the families of neonates with positive genetic results. Such testing is readily available through multiple laboratories with the same limitations due to cost, turn-around time, and difficulties in interpretation. There are many simple, low-cost biological bedside tests available able to measure both the amount and the function of the endogenous surfactant to predict RDS occurrence, which can be carried out non-invasively through the testing of blood, saliva, or buccal samples [109].

### 6.1. Quantitative Tests

Lamellar body count (LBC): Lamellar body-like particles (LBPs) can be found in lung lavages, amniotic fluids, and gastric aspirates; consider a good bedside to guide surfactant replacement therapy, as the greater the number of LBPs in those fluids, the more lung maturity [110]. The test is a quick and easy quantitative biological tool, readily available at the bedside. Nevertheless, some limitations do exist, as LBC could be unfeasible for around 35% of samples due to blood contamination and the high viscosity of many samples [111]. The test only determines pathophysiology at birth and may suggest the insurgence of RDS, rather than its clinical severity (i.e., the need for a surfactant or invasive ventilation). There is low reliability in predicting CPAP failure, as the RDS may be affected by other factors, such as the degree of alveolarization, the prenatal steroid dose, and extravascular water [110]. Furthermore, surfactant biophysical properties can be affected by the amount of surfactant protein and anionic phospholipids, the effect of reactive species of oxygen on the chemical structure of surfactant lipids and proteins, the rate of secretory phospholipase A2, and the presence of inhibitor surfactant activity substances that can be found in amniotic fluids [112].

### 6.2. Qualitative Tests

The stable microbubble test (SMT) and the surfactant adsorption test (SAT) are two quick and easy tests employed as point-of-care methods to predict CPAP failure in neonates with RDS [113].

#### 6.2.1. Stable Microbubble Test

The SMT is an old, simple, and rapidly effective method to predict RDS. The sample can be obtained from gastric aspirate, amniotic fluid, and BAL, depending on the fact that the surfactant present in amniotic fluids or gastric aspirates, when vortexed, forms numerous small stable microbubbles of at least <15 μm, which are less abundant or absent in samples from neonates with RDS [114]. Limitations of this test include that it is influenced by the subjectivity of inter- and intra-observing variability under a microscope, the efficiency to create a stable bubble is different according to the available surfactant pool inside the drops of amniotic or gastric fluid, the lack of physiopathological studies of SMT relating to lung aeration or other surfactant functions [114], and the presence of meconium in vitro may affect the stability of surfactant microbubbles tested by SMT [115].

#### 6.2.2. Surfactant Adsorption Test (SAT)

SAT is relatively quick, sensitive, not biased by any intrinsic sample dilution, and is a high-throughput fluorescent method by which to indirectly test both the adsorption and the stable accumulation of surfactant at the air-liquid interface. SAT consists of two steps: the first step includes incubation for labeling the surfactant from a biological sample, and the second step includes detection of the surfactant’s capability to move up and the kinetics of its interfacial accumulation over time [116]. SAT has been employed to assess in vitro surfactant activity from animal or cellular sources, therapeutic surfactant preparations, and non-bronchoscopic bronchoalveolar lavages (BAL) at different temperatures of asphyxiated neonates under therapeutic hypothermia. However, it is not suitable for use in clinical care, but it has been successfully used for research purposes in animals and human neonates [117].

Until now, no studies have been performed regarding SP-D levels and other surfactant proteins in different sample matrices to predict RDS in neonates. Genetic SP-D variations seem to be associated with severe RDS in very preterm birth infants [118]. Surfactant biological tests need further translational investigations and/or industrial development, so collaboration between genetic scientists, academics, and clinical practitioners is required to make progress toward the development of a quick and easy bedside test that is accurate, low-cost, and minimally invasive.

#### 6.2.3. Advanced Oxygenation Metrics

Oxygenation metrics are simple and available and solid physiopathology background tools may allow for the detection of factors influencing oxygenation, other than FiO2, and variably identify the most ill patient. Oxygenation metrics include the alveolar-arterial gradient, the oxygenation index (OI), and the oxygen saturation index (OSI).

The alveolar-arterial gradient (A-a gradient) measures the difference between the oxygen concentration in the alveoli and arterial system, and it can help to narrow the differential diagnosis for hypoxemia [119]. The a/A ratio has been used for many years using various thresholds to indicate surfactant replacement therapy. If the hypoxia is due to any pathology or dysfunction of the alveolar-capillary unit, it will result in a high A-a gradient, and if the hypoxia is due to another reason, it will result in a low A-a gradient. However, the a/A ratio is technically difficult, and ABG invasive procedures may be ethically questionable, especially in neonates with mild respiratory distress.

#### 6.2.4. Oxygenation Index (OI)

The OI is considered to be a primary indicator of respiratory disease severity in mechanically ventilated patients [120]. The OI is commonly used, as it takes the mean airway pressure into account (OI = mean airway pressure × FiO_2_ × 100/PaO_2_), and many guidelines recommend surfactant according to mean airway pressure (MAP)/positive end-expiratory pressure (PEEP), and the fraction of inspired oxygen-FiO_2_ needs [121]. The limitation of this index is the need for ABG to measure PaO_2_, which makes invasive procedures difficult to continuously measure.

#### 6.2.5. Oxygen Saturation Index (OSI)

The OI is modified to include a less invasive estimation of factors influencing oxygenation by using an oxygen saturation index (OSI), replacing PaO_2_ with peripheral saturations. The OSI is easy to disperse in neonatal critical care and correlates well with the OI, and the A-a gradient uses oxygen saturation, SpO_2_, instead of PaO_2_, which is noninvasive and can be continuously measured. The OSI is calculated as follows: OSI = [MAP (cmH_2_O) × FiO_2_ (%)]/SpO_2_ (%) [122]. The influence of fetal hemoglobin on the OSI is unknown, so the estimation of fetal hemoglobin may be included to correct saturation values and personalize the evaluation [123]. For future studies, we need more studies to detect the influence of fetal hemoglobin on the OSI and the ability of oxygenation metrics to predict CPAP failure.

#### 6.2.6. Functional Lung Imaging

##### Lung Ultrasound

Lung ultrasound (LUS) has been described as a useful bedside tool used for both descriptive and functional purposes suitable in neonates if clinical expertise exists for its interpretation, as LUS is an easy-to-learn, bedside, and quick technique and can be repeated in the event of any clinical change, providing the clinician with valuable information in the absence of ionizing radiation and with minimal disruption for the patient. However, LUS findings may be affected by many conditions, such as ventilator support, gestational age, and fluid intake, and pre-existing conditions, such as acute respiratory distress syndrome (ARDS) and broncho-pulmonary dysplasia (BPD) [124]. The ultrasound image described in neonatal respiratory distress syndrome (RDS) consists of interstitial syndrome up to white lung (grouped B-lines), sub-pleural small consolidations, and/or an irregular pleural line, as well as an air bronchogram together with variably sized consolidations [125]. Using ultrasound to assess lung aeration is a quicker indicator of surfactant need in detecting neonates with a greater risk of requiring surfactant or mechanical ventilation even before oxygenation criteria [126]. Nevertheless, the use of LUS is not specific to the loss of lung aeration due to primary surfactant deficiency but can also be useful in all cases of neonatal RD.

##### Echography-Guided Surfactant Therapy (ESTHER)

LUS-guided surfactant administration has already been investigated in a quality improvement project and a randomized controlled trial. LUS-guided surfactant administration improves oxygenation after surfactant dosing and reduces oxygen exposure early in life with possible secondary benefits, such as shorter invasive ventilation [127].

##### Functional Lung Imaging: Electrical Impedance Tomography (EIT)

EIT is a validated research tool used for dynamically imaging regional ventilation and lung function that depends on individualizing respiratory therapies in RDS. EIT measures the differential properties of air and liquids in tissues via a small non-adhesive electrode belt placed around the infant’s chest; the electrical activity is recorded to create an image representing the amounts of ventilation, aeration, gas flow, or perfusion within the area of the chest being imaged [128]. EIT also describes the effect of the surfactant during and after the replacement therapy and describes the effect of the surfactant on respiratory support strategies [129]. In neonates with RDS, EIT can demonstrate differences in end-expiratory lung volume at different CPAP levels [130]. EIT can detect and monitor changes in lung aeration caused by pneumothoraces, atelectasis, incorrect endotracheal tube placement, endotracheal suctioning, (minimal) invasive surfactant administration, and lung recruitment procedures during conventional and high-frequency ventilation in preterm infants [131].

EIT is an interesting tool, but it is less practical when applied in a clinical setting, and it cannot investigate predictions of CPAP failure. There is a need for more studies looking for early ultrasound patterns in the development of bronchopulmonary dysplasia, and the development of well-designed EIT hardware and software and easily applicable equipment for neonates is needed for clinical implementation.

Neonatologists should receive training in lung ultrasound, as it is accessible and easy to learn even with relatively little experience in this technique, as every NICU has an ultrasound device.

***ii.***  ***Bronchopulmonary Dysplasia***

Bronchopulmonary disease (BPD) is a form of chronic lung disease that is considered the most common complication of preterm birth, with worse long-term outcomes on cardiorespiratory and neurodevelopment with an increased risk of cerebral palsy and developmental delay [132]. This long-lasting sequel requires significant use of resources and funding, so it is essential to identify infants at high risk of developing BPD to prevent disease progression and target them for early intervention to prevent BPD and to reduce this healthcare burden because there is no magic cure for BPD. Once BPD has developed, it can only be managed to reduce the severity of BPD and reduce its complications [133]. If it were possible to predict those infants who would go on to develop BPD, a targeted and more tailored, personalized approach to treatments would be useful to reach optimized outcomes of care [134]. In this review, we discuss four novel non-invasive tools with potential developments in pulmonary research that allow for a more individualized optimization to prevent the development of BPD.

1. Pharmacogenetics and caffeine;
2. Electrical impedance tomography;
3. Electromyography of the diaphragm;
4. Volatile organic compounds.

##### Pharmacogenetics and Caffeine

Caffeine treatment for preterm infants is a standard of care treatment for a reduced risk of BPD with improved neurodevelopmental outcomes, but there are uncertainties regarding the mechanism of action of caffeine and its optimal dose [135]. Precision medicine might help to individualize the dosing of caffeine based on the genomic profile of preterm infants. Individual genomic variants and metabolomic heterogeneity are potential indicators for caffeine treatment effectiveness, as well as the risk of developing complications. Cytochrome P450 enzymes and adenosine receptors have known genetic associations with caffeine, and cytochrome P4501A2 (CYP1A2) enzyme activity is markedly reduced in preterm infants, leading to limited caffeine metabolism [136]. To determine a personalized caffeine dose, future research is needed to evaluate a genomic profile at birth and genomic variation in caffeine metabolism.

##### Electromyography of the Diaphragm

Changes in airway pressure or flow are used for the synchronization of invasive mechanical ventilation, but these parameters are not always accurate in the presence of a leak [137].

Transcutaneous electromyography of the diaphragm (dEMG) can detect and measure the activity of the diaphragm and provide objective information on the patient’s breathing effort.

Breath detection with dEMG is feasible and accurate in helping to individualize the application of respiratory support, titrate, and trigger the mode and level of respiratory support in preterm infants [136]. The extensive internal and external validation of transcutaneous electromyography of the diaphragm (dEMG) can be submitted to impact analyses in daily practice. In the future, more studies are needed, and neonatologists should receive training on dEMG output parameters to determine which provide the best information on the individual patient’s needs.

##### Volatile Organic Compounds

BPD is considered a multifactorial disease, as inflammation and growth failure are risk factors and mediators in its development; based on clinical characteristics or biomarkers, there is no prediction model with an accurate discrimination for the early detection of BPD.

In adult respiratory medicine, measuring volatile organic compounds (VOCs) has been increasingly used [136]. Exhaled breath is a prognostic and predictive test in preterm infants. The collection of exhaled breath is non-invasive, and volatile organic compounds (VOCs), which can be separated, qualified, and identified by chromatography-mass spectrometry (GCMS) analysis, can be rapidly determined using sensor technology. Many volatile organic compounds have been described in exhaled breath, which can represent metabolic processes in the host, bacterial metabolism, and organ function potential biomarkers [138].

Promising diagnostic or prognostic tools need extensive internal and external validation before their application in practice.

## 7. Persistent Pulmonary Hypertension in Newborn Infants

Persistent pulmonary hypertension of the newborn (PPHN) is a severe clinical problem among neonates, which significantly affects their morbidity and mortality [139]. PPHN results from a failure in the normal transition of circulation after birth and the persistent elevation of pulmonary vascular resistance (PVR), which causes right-to-left shunting, inadequate blood flow to the lungs, and severe hypoxemia. The incidence of PPHN is about 2–6 per 1000 of live births, and with a mortality rate within the range of 10–20% [140]. PPHN is multifactorial and has genetic-based susceptibility and individual risk factors. By understanding the genetic bases and risk factors associated with this condition, precision medicine can help in developing targeted prevention and treatment strategies [141]. Identification of genetic factors in the development of PPHN.

The genetic causes of persistent pulmonary hypertension are complex and involve multiple genes that regulate vascular function and response. The BMPR2 gene: This gene is crucial for regulating cell growth in the pulmonary arteries, and its mutations lead to abnormal cell proliferation, contributing to increased vascular resistance, and have been related to the familial and idiopathic elevation of pulmonary arterial pressure [141]. Other genetic variants, including those in SMAD9, NOTCH3, and CPS1, are also associated with an increased risk of PPHN [139]. The EDN1 gene, which encodes endothelin-1 (a potent vasoconstrictor), has been linked to PPHN. A specific variant (rs2070699) in this gene was found to be more prevalent in PPHN patients and associated with higher levels of endothelin-1 in the blood, suggesting its role in the pathophysiology of the disease [142]. Genetic polymorphisms and variations in genes related to pulmonary vaso-reactivity and endothelial function may influence susceptibility to PPHN [140].

### 7.1. Identification of Risk Factors for PPHN

Many factors are associated with an increase in the risk of developing PPHN, including maternal health problems, such as obesity and diabetes mellitus, and a high maternal BMI is linked to an increased risk of PPHN. The use of certain medications (e.g., serotonin reuptake inhibitors) during pregnancy has been associated with higher risks of PPHN. Perinatal conditions, such as cesarean section delivery, have a significantly higher risk of developing PPHN compared to vaginal delivery. Meconium aspiration syndrome and congenital diaphragmatic hernia are other risk factors. Sepsis, RDS, and any condition that induces hypoxia or acidosis are neonatal conditions associated with PPHN that can lead to pulmonary vascular remodeling and increase the risk of PPHN [143].

### 7.2. Deep Phenotyping

Precision medicine emphasizes the importance of deep phenotyping, which involves the comprehensive profiling of patients at the molecular level [144]. Using biomarkers for the early detection of PPHN is still ongoing in terms of research, with promising potential; for example, N-terminal pro-B-type natriuretic peptide (NT-proBNP) may be increased in PPHN and have a positive correlation with the severity of pulmonary hypertension. Endothelin-1 might be elevated in PPHN and could be used as a potential biomarker for the early recognition of PPHN [145]. Assessment of the metabolome of lung tissue or intracellular matrix can be easily carried out by sampling blood, urine, saliva, or even exhaled air. Exhaled air can be transformed into a “breath print”, assessed with the use of an “electronic nose”, for non-invasive analysis of metabolic changes in PH. The metabolomics part that can be traced in breath is called “volatolome”, and it consists of volatile organic compounds (VOC) [146]. Plasma asymmetric dimethylarginine might have diagnostic and prognostic value for predicting newborn infants who develop pulmonary hypertension. It may have diagnostic and prognostic value [147].

### 7.3. Immune Profiling and Data Integration

Recent studies have utilized machine learning to identify immune sub-phenotypes in patients with pulmonary arterial hypertension (PAH), which is closely related to PPHN. By understanding immune responses, the integration of clinical data, lifestyle factors, and environmental exposures, we construct a comprehensive health profile. This holistic view can inform treatment decisions and improve patient management [144].

### 7.4. Advanced Monitoring Techniques

Employing advanced monitoring techniques, such as electrical impedance tomography (EIT), can provide real-time data on lung function and help tailor respiratory support to individual needs. This technology allows for the better assessment of lung aeration and ventilation, which is crucial in managing infants with PPHN [136].

### 7.5. Challenges and Limitations of Precision Medicine in PPHN

Precision medicine is a promising advancement in the management of PPHN in neonates, but it faces some limitations in applications of the genetic and biological complexity of PPHN in its presentations, and its underlying mechanisms make it difficult to identify universal genetic signatures effectively. The challenges of biomarker development, considering the many potential biomarkers identified by researchers, have not yet been validated for clinical applications [146]. The methodological limitations of recent studies include the sample size and the lack of standardization in testing protocols [146]. The risk of overfitting in machine learning approaches has been used to identify patient subgroups. The need for the training and education of health care providers slows the implementation of precision medicine strategies [144].

Recommended strategies for precision medicine in neonatal persistent pulmonary hypertension (PPHN) focus on the following:

Promotion of genetic and molecular profiling [148]. Biomarker development, through research into biomarkers that correlate with disease severity and treatment response, can facilitate early diagnosis and personalized management [149]. Making individualized treatment plans and tailored pharmacotherapy based on individual responses can enhance treatment efficacy. For example, while inhaled nitric oxide (iNO) is a standard treatment, some infants may not respond adequately. In such cases, alternative therapies such as sildenafil or prostacyclin analogs can be considered based on the patient’s specific condition and response to initial treatments [149]. Establishing multidisciplinary teams that include neonatologists, cardiologists, geneticists, and pharmacologists can enhance the management of PPHN. This collaboration ensures that all aspects of the infant’s health are considered when developing a treatment plan [148]. Continued research into the genetic and environmental factors influencing PPHN is essential for developing more effective precision medicine strategies [136]. By implementing these strategies, healthcare providers can enhance the management of neonatal persistent pulmonary hypertension, leading to improved outcomes for affected infants.

The integration of precision medicine into the management of neonatal persistent pulmonary hypertension represents a significant advancement in neonatal care. Focusing on individual profiles, such as genetic and molecular insights, healthcare providers can enhance diagnostic accuracy, tailor treatments, and ultimately improve patient outcomes.

## 8. Neonatal Sepsis

Neonatal sepsis is a cause of morbidity and mortality, especially in very low-weight and preterm infants [150,151]. Understanding the heterogeneity and dynamic nature of sepsis regarding the neonatal response [151], as well as the three parts of the pathophysiological process of neonatal sepsis (genetic factors and coagulation and fibrinolysis systems), is considered a new pathway in early diagnosis, predicting prognosis, and adapting the choice of treatment to evolve from a “one-size-fits-all” to a more personalized and tailored approach to reach the optimization of the outcome of care. The gold standard for the diagnosis of neonatal sepsis is a blood culture or another sterile fluid culture in addition to sensitivity tests of the organism to antibiotics [152].

Blood culture in neonates faces multiple challenges, such as the difficulty in drawing a sufficient amount of blood for the culture, particularly in preterm infants, and neonates also have low levels of bacteremia, which requires up to 5 days to attain a positive result, and so blood culture results are delayed. Furthermore, a blood culture may not give positive results due to infection with viruses, fungi, or anaerobes, as there are no commercially available anaerobic blood culture bottles for blood volumes less than 3 mL; furthermore, the use of maternal antibiotics may lead to reduced sensitivity in the blood culture [153]. The organisms that cannot easily be cultured under available conditions are called “microbial dark matter” [154].

The current biomarkers used for sepsis diagnosis, e.g., c-reactive protein (CRP), interleukin-6 (IL-6), and procalcitonin (PCT), have achieved partial success because most of these biomarkers are associated with inflammation and are not specific to infection [155].

New diagnostic markers may be needed for the early diagnosis of sepsis, e.g., apelin, which is an endogenous ligand of the G protein-coupled receptor. APJ has been proven to compensate for the limited sensitivity and slow speed of the traditional method and is specific to infection [156]. Genetic variations in genes involved in bacterial-induced cellular response and those involved in the pathogenesis of sepsis can allow for the development of new diagnostic tools and accurate predictors of patient outcomes.

Sepsis and immune response have a genetic component, as 10% of human genes can code for mediators. Studies have implicated many genes across a number of immune and coagulation proteins, including interleukins and fibrinogen [157].

Novel molecular methods for the early detection of neonatal sepsis based on genetic and epigenetic factors support the implementation of precision medicine in neonatal sepsis. We focus on molecular diagnostic techniques with evidence for application in clinical translation, not a theoretical foundation that involves evaluating sensitivity, specificity, and accuracy to enable the early detection of neonatal sepsis.

- PCR;
- microRNA (miRNA);
- T2 magnetic resonance (T2MR) technology;
- Bioinformatics analysis.

### 8.1. PCR Techniques

PCR techniques have potential advantages in both the diagnosis and management of neonatal sepsis. These techniques are primarily based on PCR amplification techniques for the detection of 16S or 23S rRNA genes that accurately pinpoint the specific bacteria and the 18S rRNA gene of fungi [158]. Various novel PCR techniques are available (16S rRNA PCR testing, molecular multiplex PCR, molecular culture, or the sequencing of the bulk DNA and/or RNA) to identify microorganisms. These novel techniques have many advantages. They can detect small quantities of a variety of pathogens (bacterial or fungal) and DNA or RNA, identify species not easily cultured, and provide rapid results within hours. However, there are limitations, as these techniques cannot replace blood cultures and present challenges, such as the need for specialized laboratories and personnel, false-positive results due to contamination, high costs, and no available information regarding antibiotic susceptibility [159]. Quantitative PCRs can only identify resistance genes specifically targeted in the assay, but any novel mutations responsible for antimicrobial resistance remain undetected [154]. In the future, a large number of studies are still needed, as well as training clinicians in sterile sample-collecting techniques and developing more specialized laboratories.

### 8.2. MicroRNAs in Neonatal Sepsis

MicroRNAs (miRNAs) are short non-coding RNAs about 22 nucleotides in length, responsible for the regulation of gene expression by inhibiting the translation or transcription of target mRNAs that play a regulatory role in inflammation, immunity, apoptosis, and cell differentiation. More than 2000 circulating micro RNAs have been identified in the human genome that help in early diagnostic strategies for neonatal sepsis [160]; the expression of circulating miRNAs is regulated in the early stages of sepsis with a positive correlation with disease severity and progression, so blocking pro-inflammatory effects can effectively improve the related organ damage caused by sepsis [161]. miRNAs have many advantages, as sample collection is faster and less invasive, more stable in human specimens, and offer reliable measurements of expression levels. However, today, research on miRNA remains limited, and it is not possible to conduct specific studies on the subtypes of pathogens. Currently available molecular assays have insufficient diagnostic accuracy to replace microbial cultures. However, they could prove valuable, particularly in conjunction with clinical judgment and routine laboratory parameters. They can be used to help in clinical decisions and to overcome some difficulties in blood culture. In the future, a large number of studies are needed for study validation and to explore the diagnostic effect of a single miRNA on diseases.

### 8.3. T2 Magnetic Resonance (T2MR) Technology

T2MR is a novel molecular assay technology that utilizes magnetic resonance technology for direct detection and identifying the presence of pathogens in the bloodstream without the need for blood culture. These nano diagnostic panels have both T2 bacteria and T2 candida panels and demonstrate a significant advantage over blood cultures in terms of the time taken for identification while ensuring consistent sensitivity and specificity [162].

T2MR first amplifies microbial DNA by PCR, enabling detection through the resulting change in the T2 signal of the sample after probes enriched by superparamagnetic nanoparticles hybridize to the amplicon [163]. T2MR can identify circulating pathogens (either free or white-cell encapsulated), thus avoiding false-positive results associated with freely circulating DNA [164].

There are several advantages, such as rapid identification compared to blood cultures, cost-effectiveness, and suitability for development as a point-of-care diagnostic test, providing reliable support to confirm or exclude neonatal sepsis. Nevertheless, there are limitations, as the performance of T2 bacteria must be placed in context with a blood culture to enhance the diagnostic process, with potential complementary roles using each method. Most prospective studies are small or have limited numbers of comparative blood cultures, so integration between T2MR and blood cultures could help overcome current diagnostic issues, even at the most extreme ages, where clinical vulnerability requires a rapid and sensitive approach [165]. In the future, larger-scale studies and investigations for conclusive confirmation and validation are required for their potential application in neonatal sepsis.

### 8.4. Bioinformatics Analysis

Gene expression profile analysis from public databases in neonates with sepsis to develop a genetic model for predicting sepsis could provide insight into early molecular changes and biological mechanisms of neonatal early onset sepsis (EOS). Four genes, CST7, CD3G, CD247, and ANKRD22, were identified that most accurately predicted neonatal EOS and were subsequently used to construct a diagnostic model. This diagnostic model can differentiate between neonatal EOS and normal infants [166]. Meanwhile, the individual and geographic variability of EOS infants may affect the performance of this model, and the small sample size limits the validation of the model. In the future, multicenter, randomized, controlled studies are needed to evaluate the expression changes of the four genes in blood to determine if the four-gene signature identifies neonatal sepsis with negative blood cultures or not.

## 9. Renal Diseases

Precision medicine in neonatal acute kidney injury (AKI) is a research approach aimed at optimizing the management of AKI in neonates based on genetic, molecular, and environmental factors [167]. AKI is defined by a rapid reduction of the GFR, with the build-up of products of protein metabolism, the disturbance of electrolyte homeostasis, and fluid balance resulting in elevated serum creatinine levels and/or decreased urine output [168]. The management of neonatal AKI faces many challenges due to the specific physiological characteristics of neonates and their vulnerability to kidney injury from various factors, such as prematurity, infections, ischemia, nephrotoxic drugs, and metabolic imbalances. Precision medicine in neonatal AKI aims to identify at-risk infants early, understand the pathophysiology of renal injury, and identify interventions to improve outcomes [167].

Here are some key aspects of diagnostic markers of AKI in neonates:

Serum creatinine (SCr) is considered the “gold standard” of biomarkers for the diagnosis of AKI, but there are numerous obstacles with using SCr as an indicator of AKI. Most importantly, early serum creatinine in neonates could be a reflection of serum maternal levels and normalizes within days depending on the gestational age. In addition, SCr serves as a functional marker, rather than an injury one [169]. Moreover, SCr can increase (up to 48–72 h) from the renal insult and may remain at a normal level, even after the loss of 25–50% of the kidney functions [170]. These impediments interfere with the early detection of AKI, necessitating more effort to discover novel biomarkers that could accurately detect AKI, improve clinical approaches, and promote satisfied outcomes [171].

Cystatin C is a 13-kDa (kDa) cysteine proteinase inhibitor that is formed at a steady rate by all nucleated cells and is continuously secreted into the blood. It is freely filtered (>99%) in the glomeruli and then reabsorbed and catabolized in the proximal tubules. It represents a novel marker of glomerular injury, as it is not secreted by renal tubules [172]. Despite being independent of muscle mass, it has many significant limitations regarding its level monitoring, as it can be affected by many factors such as age, sex, hypertension, cholesterol levels, thyroid disease, and some medications (steroids) [173].

Neutrophil gelatinase-associated lipocalin (NGAL) is a protein produced in renal epithelia and leukocytes in response to tubular injury and systemic inflammation [173]. It has been widely studied and is now considered a strong predictive marker of AKI in heterogeneous groups of critical illnesses, even before changes in serum creatinine levels [174]. For example, NGAL was clinically applied for the detection of hypoxic-ischemic AKI [175]. In addition, children who developed post-cardiac surgery AKI have rising levels of NGAL in their urine and serum [176]. Furthermore, it was discovered that preterm babies with AKI, those who had high urinary NGAL concentrations, were distinctly associated with fatal outcomes [177]. Finally, using a cut-off of ≥400 ng/mL, NGAL in urine was significantly increased in those neonates who subsequently developed severe AKI after receiving nephrotoxic medication in the NICU [178]. Nevertheless, the NGAL assay has some limitations, as it is still not approved by the FDA, and there is no cutoff point or standardized assay specific to it [179].

Kidney injury molecule-1 (KIM-1) is a type I transmembrane protein that is elevated in response to tubular injury and may serve as a sensitive marker in neonatal AKI, especially proximal tubular insult [178].

TIMP-2 and IGFBP7: Other structural biomarkers of renal injury are tissue inhibitor metalloproteinase 2 (TIMP-2) and insulin-like growth factor binding protein 7 (IGFBP7), which are markers of cell cycle arrest. Physiologically, if this arrest was temporal, it would allow damaged DNA to be repaired and recovered. However, if cell cycle arrest persistently continues. It will cause cellular fibrosis and subsequently develop AKI early and CKD later on [180]. Therefore, these biomarkers are considered powerful indicators of preinjury, which is called acute kidney stress.

Interleukins are considered potent biomarkers in predicting AKI and play a fundamental role in its pathophysiology. Due to its anti-inflammatory role, interleukin-10 allows for the inhibition of the secretion of the proinflammatory cytokines, hindering the healing process after kidney injury [181]. In addition, studies have shown that interleukin-18 (IL-18) is linked to AKI, inducing acute tubular necrosis, and thus rising levels of interleukin-18 could be used as a risk factor for AKI [181].

Beta2-microglobulin (B2mG) is a single-chain, low molecular weight (MW = 11.8 kDA) peptide. The small structure of B2mG facilitates its rapid filtration through the renal glomeruli. However, most of the filtrated β2-microglobulins are reabsorbed and catabolized by renal proximal tubular cells. Only trace amounts of β2-microglobulin are excreted in urine. B2mG has been studied as a susceptible biomarker for AKI, being independent of muscular mass, and its rapidly rising level during renal insult gives it merit compared to serum Cr levels [181]. The rising level of serum B2mG indicates a glomerular insult, whereas tubular disorders are detected when urinary β2-microglobulin is elevated [182].

Limitation: One of the pitfalls during measuring those biomarkers is that the exact time of injury to the kidneys cannot be detected. Another limiting factor is that some of these markers are not specific to renal tubular cells but may be produced by other cells, especially in response to infection or inflammation, and can appear in the urine when they exceed the reabsorptive capacity of the renal tubules, causing a false positive renal injury diagnosis. Moreover, other laboratory abnormalities may interfere with the accuracy of their levels. For example, higher CRP and WBC and decreased serum albumin are associated with increased levels of cystatin C. In addition, albuminuria > 3000 mg/dL causes an invalid TIMP-2*IGFBP7 test [173]. In addition, the lack of feasibility of testing kits, reference standards, high costs, and variability in assay techniques and results creates more obstacles for these biomarkers to be clinically applied.

### Genetic Factors and Risk Stratification

Genetic factors play a significant role in neonatal AKI susceptibility, and precision medicine seeks to identify genetic variants that predispose neonates to kidney injury.

- Genetic susceptibility: Genetic factors have been shared in the susceptibility and severity of AKI, distinctly explaining variable AKI manifestation and different patient responses to the treatment. For example, genetic polymorphisms in APOL1 or genes related to kidney development may influence how neonates respond to hypoxia or other insults [183]. Moreover, polymorphisms in inflammation-related genes may increase the vulnerability of an individual to AKI. For example, tumor necrosis factor-α (TNF-α) [184] and nuclear factor kappa beta 1 (NFKB1) gene variants may affect the proinflammatory cytokine reaction, causing more renal damage, demand for renal replacement therapy, and in-hospital mortality [185].
- Genome-wide association study (GWAS) is a mapping method in the identification of genotype-phenotype association and novel disease susceptibility genes in an unbiased manner [186]. Furthermore, it helps in detecting the ethnic variation of complex traits, among others. This method studies the entire set of DNA (the genome) of a large group of people, searching for small variations called single nucleotide polymorphisms (SNPs) [187]. Bhatraju et al. studied nine variants determined to be associated with AKI susceptibility and reported two variants most strongly associated with AKI mapped to the DISP1-TLR5 locus [188]. Researchers hope that future genome-wide association studies will identify additional SNPs associated with AKI.
- Pharmacogenetics: Pharmacogenetics in neonatal acute kidney injury (AKI) is an emerging field that examines how genetic variations affect drug responses in neonates, particularly those at risk for or suffering from AKI. Prior to renal excretion, most of the applied drugs undergo extensive metabolism by the cytochrome P450 (CYP450) enzyme family. It is well known that the metabolic activity of CYP enzymes differs among individuals due to many factors, one of them being genetic variations. The genetic variations in CYP enzymes can cause a different metabolic activity, resulting in a different response to specific drugs metabolized by these CYP enzymes. For example, a lower metabolic activity of CYP enzymes can lead to side effects from the drugs metabolized by this enzyme, or less activity in the case of a prodrug that needs activation by this enzyme. On the other hand, the higher metabolic activity of CYP enzymes can lead to less or even no effect of drugs metabolized by this specific enzyme [189]. Genetic polymorphisms of drug-metabolizing enzymes can categorize the population according to their ability to achieve specific drug biotransformation reactions [190]. Therefore, determining a pharmacogenetic profile through pharmacogenetic studies should be augmented to optimize drug therapy, minimize adverse effects, and improve outcomes in neonatal AKI.
- Epigenetic modifications: Epigenetics is the study of the inherited factors that affect gene expression, causing changes to a phenotype without altering the DNA sequence itself [191]. Epigenetic factors, such as DNA methylation, histone modification, and non-coding RNAs, could influence the expression of genes involved in renal function, inflammation, and fibrosis, potentially contributing to AKI susceptibility or recovery. Emerging evidence suggests that epigenetic modifications could serve as biomarkers for the early detection of AKI in neonates, offering the potential for non-invasive monitoring. In addition, understanding the epigenetic landscape of neonatal AKI could achieve personalized therapies and preventative measures that target specific epigenetic modifications, helping to reduce the incidence of AKI and improve outcomes in neonates [192].

Limitation: There is a gap in the knowledge regarding the application of the genetic susceptibility of AKI, in that the small sample size reduces the statistical strength and ability to determine genetic associations. In addition, the long-term prognosis of AKI remains uncertain owing to the complex interplay with non-genetic factors.

Near-infrared spectroscopy (NIRS): Non-invasive monitoring of renal tissue oxygenation using near-infrared spectroscopy (NIRS) is a promising bedside method for the early recognition of circulatory compromise, as well as the detection of specific kidney injury and its associated morbidity and mortality [193]. Several studies have demonstrated consistent monitoring of renal tissue oxygenation in specific populations of infants, such as neonates with intrauterine growth restriction [194], infants who underwent packed red blood cell transfusions during their hospital stay [195], and neonates on ECMO for cardiorespiratory failure [196].

Early change in renal oxygenation detected by NIRS could be a unique diagnostic tool for renal injury ahead of current renal markers, allowing for earlier interventions to prevent or reduce kidney injury in various clinical conditions in the neonatal intensive care unit [193].

Gap of knowledge: The complex relationship between oxygen delivery and oxygen extraction by the kidneys, as well as concurrent cerebral and systemic oxygenation, has not been well clarified. Moreover, changes in renal perfusion may be reflected by alterations in renal tissue oxygenation, but the extent to which these hemodynamic changes impact renal function may be quite variable [192].

The accurate and precocious diagnosis of AKI is required to detect AKI risk factors and diagnosis, and therefore a personalized therapeutic strategy and follow-up plans can be established. Further studies are required using larger pediatric populations to assess the role of novel biomarkers and genetic susceptibility factors in different subtypes of AKI. In addition, reference standards for renal biomarkers should be settled to achieve their clinical application. Finally, we suggest the integration of precision medicine into clinical practice to triage patients and optimize the timing and type of interventions designed to improve disease progress and patient outcomes.

## 10. Hyperbilirubinemia

Precision medicine is an increasingly recognized approach in the management and diagnosis of neonatal hyperbilirubinemia, a common NICU problem affecting 60% of term and 80% of preterm infants [197]. Hyperbilirubinemia is either unconjugated hyperbilirubinemia or conjugated hyperbilirubinemia. Physiological hyperbilirubinemia is a transient and self-limited condition resolved in 7–10 days; however, pathological hyperbilirubinemia, when total serum bilirubin exceeds the 95th percentile for postnatal hours, is a condition that needs proper diagnosis and treatment to avoid serious neurological complications, including bilirubin-induced neurologic dysfunction (BIND) and hearing loss. Early identification and intervention are crucial to prevent long-term damage [197,198]; the risk for developing severe hyperbilirubinemia is multifactorial and has genetic-based susceptibility, as well as intra- and extra-uterine exposures [199]. Understanding these genetic types is crucial for diagnosing and managing neonatal hyperbilirubinemia effectively because it can influence treatment decisions and the need for interventions such as phototherapy or exchange transfusion [199].

Precision medicine approaches in the management of neonatal hyperbilirubinemia can involve the following aspects:

1. Genomic insights: The great advance in genomic projects has helped in the rapid sequencing and identification of genetic conditions [200].
2. Genetic testing: Identifying genetic variants associated with genetic conditions, such as UGT1A1 gene testing, or enzymes, such as glucose 6 phosphate dehydrogenase [199].
3. Diagnostic algorithms: Current diagnostic pathways are evolving to incorporate genetic testing earlier in the evaluation process, especially in conditions that need rapid interventions, such as biliary atresia.
4. Clinical assessment: Assessing individual risk factors, such as blood type and RH incompatibility, prematurity, dehydration, or breastfeeding practices, that can increase the risk of developing significant jaundice.
5. Biomarker analysis: Analyzing specific biomarkers, such as the levels of unconjugated bilirubin, reticulocyte count, and Coombs tests, can help predict the likelihood of severe hyperbilirubinemia and guide treatment decisions.
6. ML and data integration: The integration of ML algorithms with clinical data obtained from prenatal screening and genetic analysis with postnatal diagnostic work. This comprehensive data collection can lead to more accurate risk assessment and treatment [47].
7. Advancement in diagnostic technologies: The use of non–invasive bilirubinometry alongside visual assessment to improve the accuracy of diagnosing hyperbilirubinemia; this dual approach can help in making more informed treatment decisions [47].
8. Individualized treatment plans and emerging therapies: Tailoring interventions such as phototherapy or exchange transfusion based on a newborn’s genetic predisposition, risk factors, and response to initial treatments can optimize outcomes and reduce the risk of complications [201,202].

Hierarchical management and individualized treatment for neonatal hyperbilirubinemia include:

1. Tailored interventions: Treatment should be individualized based on the infant’s specific risk factors, bilirubin levels, and response to initial treatments [202].
2. Phototherapy is the first-line treatment for managing elevated bilirubin levels. The intensity and duration of phototherapy should be adjusted based on the infant’s response and bilirubin levels [203].
3. Exchange transfusion: In cases of critical hyperbilirubinemia, exchange transfusion may be necessary. The decision should be based on the infant’s clinical condition and bilirubin levels exceeding established thresholds [204]
4. Supportive care: Ensure adequate hydration and nutrition, particularly in breastfeeding infants, as breastfeeding can influence bilirubin levels and encourage home monitoring and parent care [205].

Role of audiometric brainstem response (ABR) testing:

- ABR testing is a non-invasive method used to assess the auditory pathway and detect potential hearing loss in infants with hyperbilirubinemia. It measures the brain’s response to sound stimuli and can identify abnormalities in auditory processing [206].
- Studies have shown that elevated bilirubin levels can lead to significant changes in ABR patterns, such as prolonged latencies and increased thresholds, indicating potential auditory pathway damage [207].
- By integrating ABR testing into the management of hyperbilirubinemia, healthcare providers can personalize treatment plans based on the infant’s specific risk factors and ABR results. For instance, infants with abnormal ABR findings may require closer monitoring and earlier intervention [206].

The application of individualized medicine principles in managing neonatal hyperbilirubinemia can enhance the accuracy of risk assessment, allow for prediction, early diagnosis, and the ability to tailor a person-specific intervention plan based on specific genetic information and improve long-term outcomes.

### Limitations and Challenges

Despite the promise of precision medicine to improve the management of neonatal hyperbilirubinemia, it faces several challenges, such as implementation gaps, as the field is still catching up [201]. ML data and electronic health records of genetic databases are still insufficient due to a lack of family surveys and screening approaches for genetic databases focused on neonates [200]. Limited evidence exists for the targeted intervention of the efficacy and safety of specific medications tailored to individual neonates with hyperbilirubinemia [47]. Limitations exist in terms of the resources and acceptability, which limits their application, especially in source-limited areas, and there is limited long-term follow-up for cases beyond the neonatal period for the development of hemolytic anemia, splenomegaly, or impaired liver functions to follow the course of the diseases in infants with suspected risk for genetic diseases with neonatal hyperbilirubinemia [47].

The recommended strategies for precision medicine in the field of neonatal hyperbilirubinemia are to promote monitoring and surveillance through large-scale and diverse genetic databases.

Enhance risk assessment protocols and develop comprehensive risk assessment tools that combine clinical clues with genetic information [199,208]. Regular follow-up ABR testing can help track changes in auditory function over time, providing valuable information for adjusting treatment plans as needed. This is particularly important for preterm infants who are at higher risk for both hyperbilirubinemia and hearing loss [206].

Promote family surveys and genetic counseling to form screening approaches and algorithms. Educate healthcare providers on the latest evidence-based practices that depend on individual genetic bases and environmental factors. Promote further research on the genetic and environmental factors influencing neonatal hyperbilirubinemia and promote the sharing of data among healthcare institutions [199,208].

## 11. Precision Drug Therapy in the NICU

Pharmacogenomics, Pharmacogenetics, and the Diagnostic Need for Precision Drug Therapy

One crucial aspect of neonatal care in the NICU is to provide meticulous therapeutic strategies and effective medication. The selection of drugs, as well as the determination of the dose, interval, route, and concentration, is one of the daily hard tasks regarding newborn infants with complex presentations. Newborn infants, particularly preterm and those with a very low birth weight, have immature pharmacokinetic mechanisms for drug absorption, distribution, metabolic capacity, and excretion that, together with the nature of the diseases, will adversely affect the final drug concentration in the blood. Subsequently, the therapy may or may not be effective. Moreover, drugs may cause adverse events from either toxic doses or undertreatment [209]. Furthermore, the drug doses should be monitored and adjusted to the changes in weight and postnatal age and the prospective maturation of the drug metabolism and elimination [210]. The achievement of good outcomes among sick newborn infants requires recognizing the complicated relations between the medication used, patient characteristics, and the nature and stage of illness. Identifying the metabolic phenotypes for individuals (a combination of haplotypes) assists in the selection of drug therapy and the determination of the appropriate dose according to the metabolic rate and the potential toxicity [211,212]. To implement precision drug therapy, it is important to recognize the ontogenesis process of the drug metabolism and its associated enzymes, transporters, receptors, and hepatic and renal maturity. Some disarrays might occur during the organogenesis of the liver and kidneys, which is related to their size and function, the capability of the isoenzymes, the glomerular filtration rate (GFR), and the renal tubular transport activity, as well as hemodynamic changes and blood flow. Drugs, such as ACE inhibitors, angiotensin receptor blockers, beta-lactam antibiotics, and nonsteroidal anti-inflammatory drugs, depend upon the organic anion transporters (OATs). All these factors may alter the drug concentrations during the neonatal period [210,213]. Potential drug toxicity may arise due to the slow elimination of some drugs or decreased transporter expression or GFR in the neonates that influence the excretion of penicillin, furosemide, and aminoglycosides from the kidney [214]. Immaturity/prematurity impacts the drug response; the low levels of binding protein in newborns lead to higher intracranial concentrations of the drugs than in children and adults, which potentiates the risk for drug overdose [210].

Another important aspect during neonatal and infant periods is a dynamic progressive relationship between metabolic ontogeny and pharmacogenomic aspects. The age has to be considered, as well as genetic variations, when the drug response is evaluated; e.g., morphine displays age-related extraction due to age-related rises in OCT1 and UGT2B7 protein and hepatic blood flow [215]. Also, newborn infants display unique hepatic drug metabolism compared to adults, owing to variations in P450 expression. The mechanisms controlling gene expression and induction in neonates differ from those in adults.

The activity of the P450 expression affects the hepatic drug metabolism; the CYP-dependent metabolism in neonates is 50–70% that of adults [216]. The CYP2C9 activity and CYP2D6 are very low at birth and increase during the first year of life [217]. CYP450 has a role in the metabolism and detoxification of exogenous xenobiotics and the disintegration of the majority of drugs. Phenytoin, which is commonly used for the treatment of seizures, needs to be adjusted in terms of dose in neonates depending on the CYP2C9 activity [218]. Furthermore, (CYP)2C8*3, CYP2C9*2, and CYP2C9*3 polymorphisms have lower ibuprofen clearance [219]. Similarly, the immaturity of UGT1A6 and UGT1A9 affects the glucuronidation of acetaminophen, and UGT2B7 interferes with the metabolism of morphine [220]. It is not advisable to prescribe atazanavir therapy among those with the genotype of UGT1A1 ∗28/∗28, as it increases the risk of jaundice [221]. Studies showed that the initial proper dose of warfarin should rely on age, body surface area, and VKORC1 and CYP2C9 genotypes [222].

Precision medicine in drugs addresses and distinguishes the variations in drug responses between patients. Pharmacogenomics (PK) adopts this concept and seeks to ascertain the genetic attribution to the inconsistency of the effectiveness and toxicity of the drug. Pharmacogenomics stands for how a person’s genes/DNA influence his/her reaction to precise drugs. It involves pharmacology and genomics for specific drugs to plan for a safe drug with appropriate doses that are customized to the person’s particular genes [212,223]. Pharmacogenomics recognizes genetic variants in the drug-metabolizing genes, as well as the gene mutations. Historically, PK looked at the relationship between ordinary genetic variation and patient reactions to the drugs to detect the genes that caused therapeutic phenotypic variances; however, contemporarily, it has started to distinguish mRNAs, microRNAs, and other measures that are affected by the genetic variation [224]. Current projects of precision medicine, such as precision medicine initiatives and the 100,000 genomes, need to incorporate more neonatal clinical syndromes to assess medication for complex neonatal diseases.

Therefore, we can propose that the intent of precision medicine in pharmacogenomics is to provide a specific drug with an exact dose to specific patients whose genes are matched and subsequently minimize the adverse drug reactions. The diagnostic approach of the PK supports the value of drug precision medicine, and the progress in DNA sequencing and polymorphism depiction tools facilitates the recognition of variants with pertinent outcomes. The process starts with the detection of the genetic variants in metabolizing genes of the drug that have influenced good response or toxic effect. The recent development of high-data sequencing procedures encourages the investigation of the influence of rare variants on drug efficacy. However, these techniques are not routine in current neonatal practice. Many neonatologists do not encourage genotyping due to its high cost, and the technique is not widely available. Furthermore, a study by Ghaddar et al., 2011, showed that physicians have inadequate knowledge regarding PK tests [225]; however, a more recent survey showed that the knowledge level among the studied group was fair to good, with positive attitudes [226].

The Clinical Pharmacogenetics Implementation Consortium (CPIC) sorted the drugs into A, B, C, and D levels depending on the strength of evidence that the genes affected the response to a specific drug. One study showed that genotype-directed treatment using 12 gene pharmacogenomics (PGx-PK) minimizes the adverse drug reaction by 30% in the adult population [227]. Around 75% of neonates were subjected to more than one pharmacogenomics drug. There is a complicated association between neonate traits and pharmacogenomics drugs. Drugs with pharmacogenomic indications, such as aminoglycosides, opioids, and nonsteroidal anti-inflammatory drugs, constitute 40% of the 10 most prescribed medications for extremely low birth weight and preterm newborn infants [212]. However, there are several obstacles to the adequate use of pharmacogenetics in the NICU due to scarce data on pharmacodynamics and pharmacokinetics, as well as the immaturity of newborn infants. There is a shortage of transformations of justified pharmacokinetics models to adjusted dosing [212].

Therapeutic drug monitoring (TDM) is a traditional method in pharmacokinetics used to check the concentration of drugs showing different variations between individuals [228]. Therapeutic drug monitoring, when carried out at a particular time interval, is useful for modifications of the drug dose regimen to avoid drug overdose or toxicity. Preterm infants or neonates with acute kidney injury are vulnerable to medication adverse events. Some drugs, such as theophylline, aminoglycosides/gentamicin, vancomycin, and antifungal agents, such as itraconazole and voriconazole, have low safety margins and a narrow therapeutic index; neonates may benefit from drug monitoring to preclude ototoxicity and nephrotoxicity [212,229].

The monitoring of the concentration of drugs is ideal for those with genetic polymorphism, such as CYP2C19 monitoring [230]. One study showed major individual variances in voriconazole metabolism in children. Linking the TDM with the CYP2C19 gene polymorphism disclosed valuable knowledge for customized antifungal therapy in pediatric patients. TDM is also available for phenobarbital and phenytoin, which are the most common antiepileptic drugs to avoid considering their side effects on the developing brain of preterm and newborns [230,231,232,233]. Moreover, the use of time-division multiplexing to measure blood drug levels, such as anticonvulsants or antibiotics, optimizes the doses and prevents drug side effects that may occur even with normal doses in sick neonates with kidney or hepatic disorders. Pharmacogenomic variants aid in selecting the right drugs for specific patients and calculating the dose [17].

Therapeutic hypothermia (TH) is an important and quite recent therapy in the NICU. There are contradicting data regarding the pharmacokinetics of drugs during TH, as some studies have shown diminished drug clearance by glomerular filtration and decreased activity of hepatic cytochrome enzymes [234]. Some drugs, such as phenobarbital, showed an increase in plasma concentrations and prolonged half-lives during TH [235]. Nevertheless, other studies showed no noteworthy changes [236]. Conversely, the ParmaCool study increased the phenobarbital dose to 30 instead of 20 mg/kg to achieve curative concentrations during TH [237]. Furthermore, the modification of the doses or the duration of antibiotics during TH was advised in several studies; amikacin and gentamicin showed a decline in clearance during TH, and 36 h dose intervals were recommended. The modification of the ampicillin dose to 25–50 mg/kg/day was recommended throughout TH [238].

Introducing new approaches for prescribing therapeutic doses in newborn infants has lagged behind those in children. However, there have been recent studies for precision dosing in neonates. These approaches depend on advances in diagnostic technology, such as mass spectroscopy, pharmacogenomics in neonates, Bayesian assessment, electronic decision support means, and software that incorporates pharmacokinetics and pharmacodynamics. These tools provide pharmacokinetic-/pharmacodynamic-model-based methods that integrate population- and physiology-based pharmacology data.

These recent advances provided appropriate modifications of drug dosages to attain the required outcome without harmful effects when depending on age or body weight [239]. Physiology-based pharmacokinetic (PBPK) models merge the physiological and anatomical data of the patients, and the biochemical traits of drugs are useful to guide the dose exposure relationship and plan for the best therapeutic dose, while population pharmacokinetics modeling (PopPK) defines the time sequence of the drug experience in patients and explores the causes of differences in patient exposure; it advises the initial prescribed dose, and both models have supported our insight into drug metabolism in neonates [240,241,242].

The clinical implementation of these models has shown promising results. The precise dose of fluconazole and acetaminophen is advised by using population PK modeling [243]. Mahmood et al., 2017, used the PBPK-model-based approach to predict the elimination of glucuronidated drugs in newborn infants [244].

The PBPK has been reported to successfully include the ontogeny of drug-metabolizing enzymes involved in acetaminophen metabolism in preterm infants. It was an important finding, as acetaminophen is used in the NICU for pain control and ductus arteriosus closure. An inappropriate dose may increase the risk of hepatic toxicity [245].

Another model was described by Vicks et al., 2020, to prescribe morphine precision dosing through the utilization of the electronic health record and pharmacokinetic-model-informed dosing advice [246]. The model can integrate real-time drug concentration information and allows for a prompt response regarding a decision on the appropriate dose. Another model for determining the precision morphine dosage for the management of neonatal pain based on informed Bayesian estimation can also adjust morphine doses in neonates and infants [247].

Tong et al., 2022, showed the value of the continuous improvement of using the model-informed precision dosing (MIDP) in neonatal practice. The improvement of gentamicin MIPD in neonates has shown a reduction in the need for several blood samples for the drug monitoring of gentamicin [247].

Vancomycin has high variable pharmacokinetics because of developmental changes and grades of disease severity in neonates. Vancomycin-model-informed precision dosing software appears to be a promising and safe approach to improving the PKPD dose of vancomycin in newborn infants admitted to the NICU [248,249]. The population pharmacokinetic model, conjoined with the opportunistic sampling approach, has been shown to be a practical approach for ganciclovir precise dose in newborns with congenital CMV infection [250].

One of the limitations of relying on model-informed precision dosing is that it does not include the majority of drugs used in the NICU. Additionally, models for analgesics/morphine do not consider the nature of the painful stimuli, whether invasive procedures or prolonged events, such as mechanical ventilation. Moreover, the painful stimuli provoke the release of various chemical substances that may affect the dose of morphine [251]. Models that consider the dosage of drugs during TH are not yet available. The NICU patients are subjected to various off-label medications in the NICU and are vulnerable to adverse drug events [252]. The financial cost needs to be evaluated, and the capacity building of the physician to use these technologies is needed to implement the different models.

As we have shown above, genome sequencing is now available for the neonatal population. Combining genomic data with pharmacogenomics data will lead to better therapeutic outcomes [253]. The involvement of neonatologists and related healthcare workers to improve pharmacogenetics knowledge and clinical application of new pharmacology methods in the field of neonatology has to start worldwide [254].

To maximize the advantage of therapeutic precision medicine in neonates, more studies need to be carried out pertaining to pharmacogenetics to enhance better outcomes and diminish the expenditures from inadequate treatment. Therefore, future research needs to include the ontogeny of the drug-metabolizing enzymes, transporters, and receptors, with associated physiologic changes in postnatal age to the model-informed precision dosing in the NICU to permit precise therapeutic doses. Research involving drug interactions and genetic variations is essential to avoid adverse events among sick neonates who are subjected to multiple drugs and several therapeutic modalities.

Finally, neonatologists have to take into consideration all the recent achievements in pharmacogenomics and pharmacokinetics whenever possible and indicate the use of genotyping tests and obtain the best results from applying available model-informed precision dosing in the NICU. The PM Initiative, which seeks to leverage advancements in genome biology, next-generation sequencing, and digital health, alongside ongoing PK/PD studies, is expected to further enhance the progress achieved through improved drug therapy. Clinical trials to validate the current approaches and to ensure the implementation of ethics and equity are needed.

## 12. Conclusions

In recent years, the integration of precision medicine into neonatal care has gained momentum, supported by new research that underscores its effectiveness. The evolution of diagnostic measures is vital in managing neonatal disease, especially among neonates admitted to the NICU. Traditionally, diagnosis relied on clinical examination, observations, and basic laboratory and radiological assessments. This review explored the shift towards more refined diagnostic criteria. The development of precise diagnostic criteria offers a pathway to more effective and individualized healthcare for neonates admitted to the NICU.

The importance of this topic lies not only in its medical implications but also in its perspective to enhance the overall quality of life for these newborn infants, as timely and accurate diagnosis is essential for implementing early interventions, which can mitigate the effects of brain injury or other organ failure.

The advancements in PM, particularly in the fields of genetics, metabolomics, and ML, are revolutionizing the diagnosis and treatment of neonatal complex illnesses. For example, this evolution can distinguish between HIE and non-HIE causes of neonatal encephalopathy, as well as causes of seizures and respiratory distress syndrome, hence determining the appropriate course of treatment.

With the utilization of sophisticated algorithms and biological data, clinicians can now make more informed decisions, leading to enhanced individualized healthcare. The metabolomics can offer understanding into the biochemical changes occurring in the infant’s body, further guiding treatment decisions. The genetic analysis revealed several significant associations between specific gene variants and the risk of developing HIE, BPD, ROP, NEC, RDS, and AKI.

The implementation of noninvasive tools, such as point-of-care ultrasound, echocardiography, and NIRS, in the NICU embodies how personalized treatment strategies can be facilitated by advanced monitoring technologies. Precise prediction of hs PDA and identification of shock phenotypes help to provide and monitor individualized therapy.

The review concludes by emphasizing the transformative potential of the new advancement tools in improving outcomes for newborn infants affected by complex illnesses, such as neonatal encephalopathy and disturbed hemodynamics due to PDA or shock, respiratory distress syndrome, kidney injury, hyperbilirubinemia, PPHN, and sepsis. The review also stresses the diagnosis of aspects affecting response to therapy, such as as pharmacokinetics and pharmacogenetics. The paper further suggests avenues for future directions/research in the topics of neonatal precision medicine.

### Future Direction

We must remain committed to refining diagnostic criteria and diagnostic technology that can be implemented in daily use in the NICU, with high predictive accuracy and timeliness for early diagnosis on an individual basis. These technologies have to be affordable and user-friendly, with a reasonable cost, and ensure ethical and equitable treatment for all infants to improve the short- and long-term outcomes. In doing so, neonatologists will be able to personalize therapy and enhance future neonatal care.

Future research must enhance the diagnostic system and monitoring of newborn infants with complex and critical illnesses, incorporating clinical and biological markers, genetic profiles, metabolomics studies, and imaging tools, such as NIRS, MRI, EEG, and echocardiography, as well as using AI resources, such as machine learning algorithm models that can analyze complex datasets. For example, large-scale studies that evaluate the effectiveness of machine learning algorithms in diverse clinical settings can help validate their utility and enhance their integration into routine practice. Additionally, ongoing exploration into metabolomics biomarkers may unveil new targets for intervention, ultimately leading to more effective treatment strategies. The development of well-designed hardware and software machines for neonates is needed for clinical implementation, and neonatologists should receive training in using these techniques.

## Figures and Tables

**Table 1:** Genetic surfactant-related disorders.

Protein	SP-B	SP-C	ABCA3	SP-A	SP-D	TTF-1	GM-CSF Receptor [3,4]
Gene	SFTPB	SFTPC	ABCA3	SFTPA1 SFTPA2	SFTPD	NKX2–1	CSFR2A CSFR2B
Pulmonary Phenotypes	RDS	ILD PF RDS	RDS PPHN ILD PF	PF Lung cancer	None yet known	RDS ILD Recurrent Infection	Alveolar Proteinosis
Inheritance	AR	AD sporadic	AR	AD sporadic	N.A.	Sporadic AD	AR
Prognosis	Rapidly fatal	Variable	~60% rapidly fatal; ~40% variable	Generally adult onset, progressive	N.A.	Variable	Childhood to adult-onset; variable
Incidence	<1 in 1,000,000	Unknown	Uncertain, 1 in 10 K to 1 in 20 K	Unknown	N.A.	Unknown	Unknown

RDS, respiratory distress syndrome; ILD, interstitial lung disease; PF, pulmonary fibrosis; PPHN, persistent pulmonary hypertension of the newborn; AR, autosomal recessive; AD, autosomal dominant [108]. N.A.: Not Available.
